# NCAPH serves as a prognostic factor and promotes the tumor progression in glioma through PI3K/AKT signaling pathway

**DOI:** 10.1007/s11010-024-04976-4

**Published:** 2024-04-08

**Authors:** Jianshen Liang, Debo Yun, Wenzhe Jin, Jikang Fan, Xuya Wang, Xisen Wang, Yiming Li, Shengping Yu, Chen Zhang, Tao Li, Xuejun Yang

**Affiliations:** 1 Department of Neurosurgery, Tianjin Medical University General Hospital, Tianjin, 300000 China; 2 Laboratory of Neuro-Oncology, Tianjin Neurological Institute, Tianjin, 300000 China; 3 Department of Neurosurgery, Nanchong Central Hospital, Nanchong, 637000 Sichuan China; 4 Department of Neurosurgery, Affiliated Hospital of Hebei University, Baoding, 071000 Hebei China; 5 Department of Neurosurgery, Tsinghua University Beijing Tsinghua Changgung Hospital, Beijing, 102218 China

**Keywords:** Glioma, NCAPH, PI3K/AKT, Central nervous system

## Abstract

Non-SMC (Structural Maintenance of Chromosomes) condensin I complex subunit H (NCAPH) has been shown to facilitate progression and predict adverse prognostic outcome in many cancer types. However, the function of NCAPH in gliomas is still unclear. Series of experiments were taken to uncover the function of NCAPH in glioma. The expression of NCAPH and potential mechanism regulating progression of glioma was verified by bioinformatics analysis. Lentiviral transfection was used for establishment of loss-of-function and gain-of-function cell lines. CCK-8 assay and Colony-formation assay were used to evaluate proliferation. Transwell assay and Cell wound healing assay were used to assess migration and invasion. Cell cycle and apoptosis were measured by flow cytometry. Protein and RNA were quantified by WB and RT-PCR, respectively. The nude mice model of glioma was used to evaluate the effect of NCAPH in vivo. The expression of NCAPH increased significantly in glioma tissues and correlated with WHO grade, IDH wild-type and non-1p/19q codeletion. Glioma patients with high expression of NCAPH had an undesirable prognosis. Functionally, upregulated NCAPH promotes the malignant hallmarks of glioma cells in vivo and in vitro. NCAPH correlated with DNA damage repair ability of glioma cells and facilitated the proliferation, invasion, and migration of glioma cells by promoting the PI3K/AKT signaling pathway. This study identifies the important pro-tumor role of NCAPH in glioma and suggests that NCAPH is a potential therapeutic target.

**Supplementary Information:**

The online version contains supplementary material available at 10.1007/s11010-024-04976-4.

## Introduction

Gliomas, originating from glial cells or precursor cells, are the most common primary central nervous system (CNS) malignancies including astrocytoma, oligodendroglioma, and ependymoma [1, 2]. The World Health Organization (WHO) classifies gliomas into four grades, with grades 1 and 2 classified as low-grade gliomas (LGG) and grades 3 and 4 as high-grade gliomas (HGG) [3]. The median survival time of LGG patients is 11.6 years [4]. For HGG patients, the median survival time is about 3 years in grade 3 patients, and the prognosis of grade 4 patients was the worst, with a median overall survival time of only 15 months [5]. Isocitrate dehydrogenase (IDH) and 1p/19q status have been applied to the diagnosis of glioma [3]. Diffuse gliomas with IDH mutation confer a better prognosis than diffuse gliomas with IDH wild-type [6]. In addition, 1p/19q codeletion has a good prognosis in diffuse gliomas [7]. The last decade has seen the emergence of novel immunotherapies, including Chimeric Antigen Receptor T-Cell Immunotherapy (CAR-T therapy) [8], cancer vaccines [8–10], oncolytic virotherapy [11, 12], and monoclonal antibodies (checkpoint inhibitors) [13] that have been employed for the management of glioma patients. In addition, there are several targeted therapies which can inhibit activation of abnormal cell-signaling pathways implicated in the primary brain tumors [14]. However, none of the existing therapies could significantly extend overall survival (OS) in clinical trials. Therefore, it is urgent to explore new targeted molecules for the treatment of glioma.

Condensin I and condensin II, two types of DNA condensin complexes, involve in chromosome condensation and segregation during mitosis in most eukaryotic cells [15]. Non-SMC condensin I complex subunit H (NCAPH), located on chromosome 2q11.2, is an essential regulatory subunit of condensin I [16]. NCAPH maintains the stability of the condensin complex and ensures the precision of sister chromatids separation in cell mitosis [15–17]. NCAPH can effectively regulate the mature DNA damage and chromosome condensation [18]. Additionally, NCAPH can possibly affect development of the various tumors. For instance, by forming regenerative feedback with human papilloma virus E7 (HPV E7), upregulated NCAPH enhances the proliferation ability of cervical cancer cells through stimulating the PI3K/AKT/SGK pathway [19]. In colorectal cancer, depletion of NCAPH significantly inhibits the tumor growth and migration and induces apoptosis as well as cell cycle arrest [20]. NCAPH regulates gastric cancer progression through DNA damage response [21]. Moreover, down-regulation of NCAPH has been reported to significantly inhibit the progression of several cancer cell lines [22–24]. However, the function of NCAPH in glioma remains unclear previously.

PI3K/AKT signaling pathway is involved in the regulation of many types of cancer, and the activation of this pathway is associated with cancer proliferation, migration, invasion, and DNA damage [19, 25, 26]. Previous studies have found that when PI3K/AKT signaling pathway is inhibited, the proliferation, invasion of glioma cells will be reduced [27, 28], while DNA damage of tumor cells will be increased accordingly [29]. However, there has been no comprehensive analysis of the correlation between NCAPH and PI3K/AKT signaling in glioma cells.

In this study, we found the expression of NCAPH was upregulated in gliomas by bioinformatics analysis and was associated with poor prognosis. In addition, NCAPH was correlated with clinical features including WHO grade, IDH wild-type, and non-1p/19q codeletion. Enrichment analysis, flow cytometry, and correlation analysis revealed NCAPH promoted cell cycle of glioma cells from G1 to S phase, inhibited apoptosis, and was highly correlated with DNA damage repair genes. Subsequently, we established gain-of-function and loss-of-function cell lines and identified that NCAPH promoted glioma cell proliferation, migration, invasion, and epithelial–mesenchymal transition (EMT) progression. Mechanistically, NCAPH regulated the malignant progression of glioma cells through the PI3K/AKT signaling. Finally, the tumor-promoting effect of NCAPH in vivo was further verified by constructing a glioma nude mouse model. Taken together, NCAPH may play as a pro-tumor role and serve a potential therapeutic target.

## Materials and methods

### Bioinformatics analysis

Pan-cancer expression and clinical data were downloaded from UCSCXENA (https://xenabrowser.net/datapages/), and were sorted uniformly. NCAPH expression in glioma and clinical data were also obtained from CGGA database (http://www.cgga.org.cn). Kaplan–Meier (K-M) survival curve is used to analyze effects of NCAPH on OS of cancer patients. Then, pROC package and timeROC package were taken to perform receiver operating characteristic curve (ROC) analysis, and the results were visualized with ggplot2. The "DESeq2" package was obtained to screen the differentially expressed genes (DEGs) using R Studio. We then analyzed immunohistochemical (IHC) image from The Human Protein Atlas (https://www.proteinatlas.org/) to directly compare the NCAPH protein expression between glioma and normal tissues. We employed the "WGCNA" package in R Studio to divide DEGs into diverse kinds of gene modules and obtain blue module with the highest correlation, which facilitated extraction of the corresponding core gene set. The target gene NCAPH and the core genes of the blue module were imported in the STRING database (https://string-db.org/) to make clear the mutual relation networks of NCAPH encoded proteins and the results were visualized by Cytoscape. DAVID online database (https://david-d.ncifcrf.gov/) was used in the Gene Ontology (GO) analyses and Kyoto Encyclopedia of Genes and Genomes (KEGG) pathway enrichment analyses. After analyzing the correlation between NCAPH and DNA damage repair genes, gglot2 was used to visualize the results. Matrix scores, immune scores, and tumor purity were then quantified under a R-packet estimate. We analyzed the ssGSEA algorithm based on the R package GSVA, and used markers of 24 different immune cells mentioned in prior published article [30] to calculate the corresponding data related to the immunity infiltration situation.

### Cell lines

U118MG, U87MG, and U251MG cells were purchased from the Chinese Academy of Sciences Cell Bank (China) and LN229, LN18, A172, and T98G cell lines were obtained from ATCC (USA). TJ905 cell was separated from human GBM tissue and was cultivated in a mixture consisting of 10% fetal bovine serum (FBS, Gibco, USA) and DMEM/F12 medium (Gibco, USA). DMEM (Gibco, USA) mixed with 10% FBS was used to cultured other seven glioma cell lines which were incubated in 5% CO_2_ at constant temperature of 37 °C. Cells were identified by polymorphic short tandem repeat profiling in advance and tested every 6 months for excluding mycoplasma contamination.

### Clinical sample collection

All clinical tissues and pathologic diagnoses of glioma patients from intraoperative resection were obtained from the Department of Neurosurgery, Tianjin Medical University General Hospital, China, from August 2011 to April 2017 [31]. All samples were histologically diagnosed by pathologists according to the World Health Organization (WHO) classification. Written informed consent was obtained from all donors or their relatives. This research approved by the ethical committee of Tianjin Medical University General Hospital was performed in accordance with the principles of the Helsinki Declaration.

### H&E staining and immunohistochemistry (IHC) analysis

Paraffin-embedded tissues applied to H&E staining and IHC analysis were prepared as previously described [32]. Glioma tissues and mouse brain tissues were incubated with primary antibodies (NCAPH, 1:100, 11515–1-AP, Proteintech, USA; PIK3CA, 1:100, A0265, ABclonal, USA; Ki67, 1:100, A23722, ABclonal, USA). IHC markers were detected using a goat anti-rabbit IgG two-step detection kit (PV-9001, ZSGB-Bio, China). Next, the Mayer Hematoxylin Solution (G1080, Solarbio, China) was used for nuclear staining. The IHC staining images were acquired by a VANOX microscope (Olympus, Japan).

### Lentivirus transfection

Three shRNA-NCAPH sequences (sh-1, 5′-ccCAAGGATTAGACATCACAA-3′; sh-2, 5′- acACGCAGATTACGGAACATT-3′; sh-3, 5′-gcACCGTCTTTGGAAGAAGTA-3′) and one negative control transferred scramble sequence (5′-TTCTCCGAACGTGTCACGT-3′) were established by GV493 vector. Based on the GV492 vector (GeneChem, China), we also established an over-expressed plasmid of NCAPH, and a corresponding control transferred scramble sequence was also designed. The lentiviral transfection was performed using instructions provided by the manufacturer. After infecting, U87MG, LN229 glioma cells were screened by puromycin under 2.00 μg/ml solution. We then designed PIK3CA-overexpressing plasmids based on pcDNA3.1 and transiently transfected glioma cells through Lipofectamine 3000 (Invitrogen, US).

### Real-time polymerase chain reaction (RT-PCR) and Western blotting (WB)

All RNA and protein extraction and the following RT-PCR and WB assays were performed as previously reported [33]. The mRNA expressed-level of NCAPH and GAPDH used as internal control was examined by applying GoTaq qPCR Master Mix (A6001, Promega, USA). The primer sequences (Genewiz, China) used were as following: NCAPH: F: 5′-GTCCTCGAAGACTTTCCTCAGA-3′, R: 5′-TGAAATGTCAATACTCCTGCTGG-3′; GAPDH: F: 5′-GGTGGTCTCCTCTGACTTCAACA-3′, R: 5′-GTTGCTGTAGCCAAATTCGTTGT-3′. The relative standard curve method was employed to analyze data and GAPDH was used for normalization. The protein expression was measured by GBOX (Syngene Company, UK) after adding a chemiluminescent HRP substrate (WBKLS0500, Millipore, USA). Primary antibodies used were as following: NCAPH at 1:800 dilution (11515–1-AP) from Proteintech (USA); PIK3CA at 1:1000 dilution (A0265) from ABclonal (USA). E-cadherin at 1:2000 dilution (E-Ca; 3195S), N-cadherin at 1:1500 dilution (N-Ca; 13116S), vimentin at 1:1000 dilution (5741S), Snail at 1:1000 dilution (3879S), AKT at 1:1000 dilution (9272S), and p-AKT at 1:1000 (4060) and all these antibodies were purchased from Cell Signaling Technology (USA). GAPDH (TA-08) was obtained from ZSGB-BIO (China).

### Cell cycle and apoptosis analysis

Cell cycle and apoptosis analysis were examined by flow cytometry. For cell cycle analysis, 1 × 10^6 cells were fixed with 70% ethanol for 1 h and then were incubated with PI/RNase staining solution (CY001, Simu Biotech Co., Ltd) for 30 min. For apoptosis analysis, 1 × 10^6 cells were incubated with Annexin V-FITC and 7-AAD (A5001-03A, Simu Biotech Co., Ltd) for 15 min. Subsequently, cell cycle and apoptosis were measured by a flow cytometer (BD FACSVerse, Becton, Dickinson and Company, USA).

### Cell counting kit-8 assay (CCK-8)

Based on the manufacturer’s instructions, the proliferation of glioma cells was analyzed utilizing CCK-8 assay kit (CK04, DOJINDO, China). 2.0 × 10^3 cells were seeded in each well of 96 well plates and were cultured for 4 days in 5% CO_2_ at constant temperature of 37 °C. Before measuring absorbance, cells were incubated with CCK-8 solution for 1 h, and absorbance was measured at 450 nm using a microplate luminometer (BioTek, USA).

### Colony-formation assay

For colony forming assay, 1 × 10^3 glioma cells were seeded in a six well plate and then incubated in 5% CO_2_ at 37 °C for 2 weeks. Next, we used PBS to wash the cells, paraformaldehyde (P1110, Solarbio, China) for cell fixation, and 2.5% crystal violet stain solution (G1061, Solarbio, China) for staining the cells. The efficient quantification was measured as a proportion of the colony formation counts to the counts of the cells seeded.

### Transwell assay

As for Transwell migration assay, glioma cells (1 × 10^4 cells) were inoculated in upper chamber vessel of the 24-well Cell Culture Insert (Corning, USA) and serum-free DMEM was added, whereas 10% FBS and medium as chemoattractant was added to the lower well. After incubation for 36 h, we used cotton swab to remove the non-invading cells in the upper chamber. The migrating cells in the Transwell chamber were then fixed by 4% paraformaldehyde and stained using 2.5% crystal violet stain solution. Finally, we qualified the counts of migrating cells that fixed on the surface below the chamber.

As for Transwell invasion assay, Matrigel was overspread at the bottom firstly. 3 × 10^4 cells were seeded into the upper chamber containing the serum-free DMEM and were incubated for 48 h. The subsequent operations were performed as in Transwell migration assay.

### Cell wound healing assay

5 × 10^5 cells spread into 6-well cell culture insert. When the cell fit reached 80%, 200 μl pipette tips were used to scratch a straight line. Three straight lines were drawn in one hole, one in the middle, and two on each side. Subsequently, all wells were washed with PBS for 3 times to remove the floating cells. Serum-free medium was added and the photos were captured through the microscope. The photos of each group were recorded for 0 h, and then the cells were cultured at 37 °C in 5%CO_2_ incubator. The recording was continued for the 24th hour and the 48th hour using the microscope.

### Intracranial xenograft model in nude mice

The nude mouse model of intracranial xenotransplantation was constructed based on the findings of previous studies [32]. Eighteen nude mice were divided into three different groups: OE-NCAPH group, sh-NCAPH group, and the control group, and U87MG cells transfected with the corresponding lentivirus were injected into mouse brains. The relative changes in body weight and survival status of mice in each group were observed every day, and the tumor load was monitored by bioluminescence imaging (measured by IVIS spectral real-time imaging system (PerkinElmer, USA)) for 4 consecutive times every week from the 7th day. On the 50th day after the glioma model was established, all mice were sacrificed.

### Statistical analysis

SPSS 20 was used for the statistical analysis. All the quantitative experimental data were measured at least three times, and displayed as the mean ± SD. The log-rank (Mantel–Cox) test was used to determine the Survival analyses in GraphPad Prism 9.0. Correlation analysis was measured by Spearman. The comparison in the means of two groups was measured through an unpaired t-test, and a two-tailed *p* value of  < 0.05 was considered as statistically significant.

## Results

### NCAPH is highly expressed in 19 cancers and displays poor prognosis in 6 tumor types

**Fig. 1:**
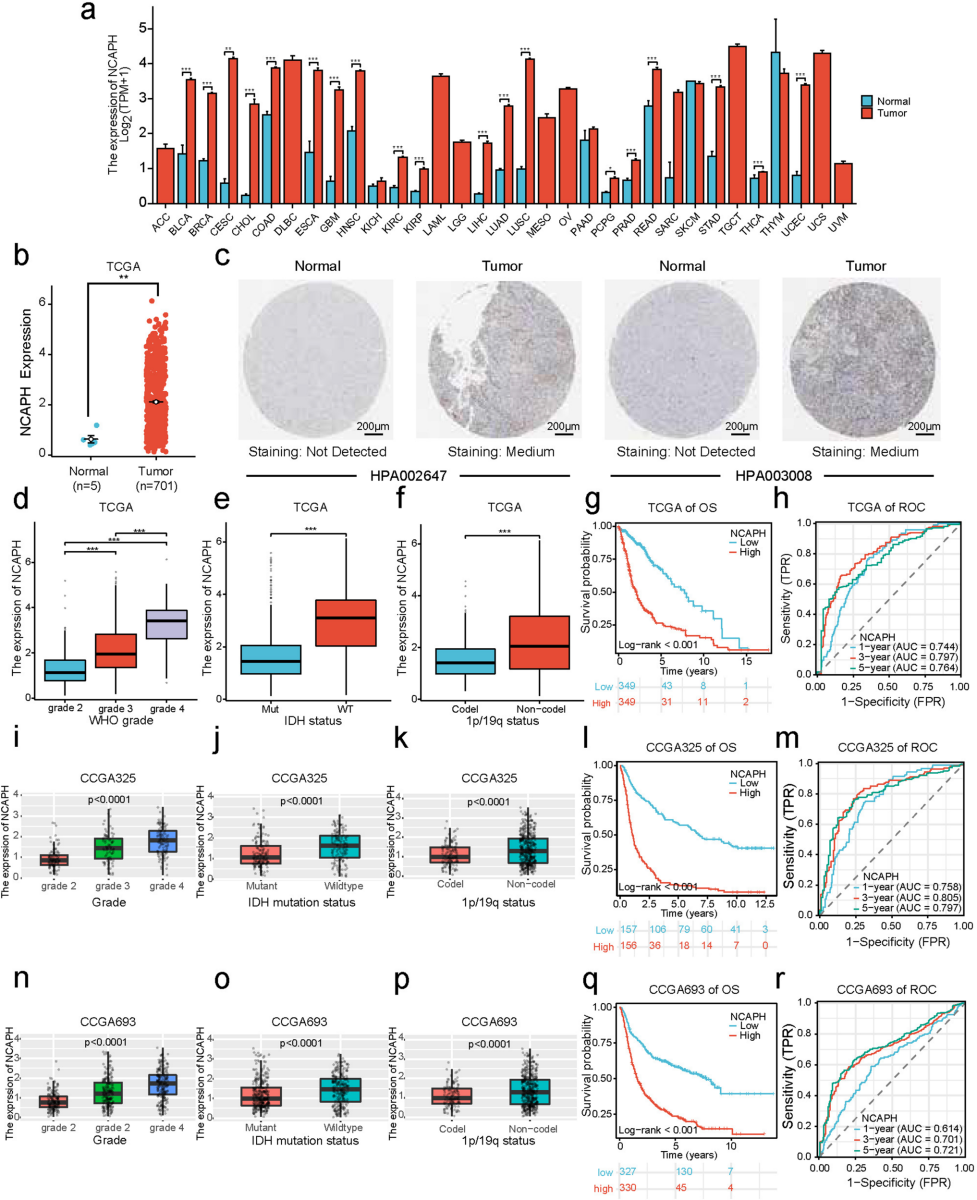
Expression of NCAPH in pan-cancer and prognostic value of NCAPH in glioma. **a** NCAPH expression in pan-cancer tissues and corresponding normal tissues. **b** NCAPH expression in glioma and normal tissues. **c** Comparison of immunohistochemical images of NCAPH between glioma and normal brain tissue. **d**,** i**,** n** NCAPH expression corresponding to WHO grade in TCGA database, CGGA325 and CGGA693 dataset. **e**,**j**,**o** NCAPH expression in glioma cells corresponding to IDH status in TCGA database, CGGA325 and CGGA693 dataset. **f**,**k**,**p** NCAPH expression derived from TCGA database, CGGA325 and CGGA693 dataset in glioma cells corresponding to 1p/19q status. **g**,**l**,**q** K–M survival analysis of NCAPH in glioma in TCGA database, CGGA325 and CGGA693 dataset. **h**,**m**,**r** The time-dependent diagnostic efficacy ROC curve of glioma response to NCAPH in TCGA database, CGGA325 and CGGA693 dataset (^ns^*p* > 0.05, *p < 0.05, **p < 0.01, ***p < 0.001, ****p < 0.0001 here and in the following figures)

Firstly, we explored the expression of NCAPH in different cancer types (Fig. 1a). As for the comparison between 33 different cancer types and their normal counterparts, the expression of NCAPH was not uniformly upregulated in all 33 cancers. It was observed that among 19 different types of cancer (BLCA, BRCA, CESC, CHOL, COAD, ESCA, GBM, HNSC, KIRC, KIRP, LIHC, LUAD, LUSC, PCPG, PRAD, READ, STAD, THCA, and UCEC), the expression of NCAPH was significantly upregulated.

For further understand the prognostic value of NCAPH in different cancer types, we combined TCGA clinical data and RNA-seq data of above 19 cancers to plot Kaplan–Meier (K–M) survival curve (Fig. S1). Before that, to explore the potential function of NCAPH in glioma more comprehensively, low-grade glioma (LGG) and glioblastoma multiforme (GBM) were combined into glioma group (GBMLGG) for analysis. The outcomes revealed that the OS time in GBMLGG (*p* < 0.001) (Fig. S1g), KIRC (*p* = 0.021) (Fig. S1i), KIRP (*p* < 0.001) (Fig. S1j), LIHC (*p* = 0.008) (Fig. S1k), LUAD (*p* = 0.006) (Fig. S1l), and UCEC (*p* = 0.024) (Fig. S1s) which displayed higher expression level of NCAPH was significantly more adverse. These results suggested that NCAPH can be an important clinical marker at least for the above six tumors.

### Upregulated NCAPH expression is associated with WHO grade, IDH wild-type, and non-1p/19q codeletion in glioma

As the potential role of NCAPH in glioma is unclear, we decided to conduct an in-depth exploration about the function of NCAPH in glioma. It was found that in TCGA database, compared to normal population (*n* = 5), glioma patients (*n* = 701) exhibited a significantly higher expression level (Fig. 1b, *p* < 0.01). Results of IHC also indicated the expression of NCAPH protein in glioma tissue was significantly higher than that in normal brain tissues (Fig. 1c and Fig. S2). Furthermore, in baseline data sheet, we observed there was no association noted with respect to patient gender (*p* = 0.9740), but the NCAPH significantly correlated with clinicopathologic parameters like patient age (*p* < 0.001), tumor WHO grade (*p* < 0.001), pathological class (*p* < 0.001), IDH status (*p* < 0.001), as well as 1p/19q status (*p* < 0.001) (Tab. S1). Meanwhile, through TCGA data, data sets CGGA325 and CGGA693, we identified the up-regulation of NCAPH correlated with WHO grade (Fig. 1d, i, n), IDH wild-type (Fig. 1e, j, o) and non-1p/19q codeletion status (Fig. 1f, k, p). Subsequently, K–M survival analysis suggested there was more adverse OS in high NCAPH group (Fig. 1g, l, q, *p* < 0.001). Besides, the expression of NCAPH was highly associated with 1-year survival (AUC = 0.744, 0.758, 0.614), 3-year survival (AUC = 0.797, 0.805, 0.701), and 5-year survival (AUC = 0.764, 0.797, 0.721) of patients, respectively (Fig. 1h, m, r). After adjusting other parameters in multivariate Cox regression analysis such as patient age, tumor grade, IDH status, and 1p/19q status, the results manifested NCAPH might play a predictor in glioma. (Tab. S2). In conclusion, the expression of NCAPH is significantly upregulated in glioma, and higher NCAPH correlates with characters of undesirable prognosis like WHO grade, IDH wild-type, and non-1p/19q codeletion.

### The blue module genes closely related to NCAPH are screened by WGCNA

To further analysis, 2086 DEGs between glioma and normal tissues were selected from TCGA and weighted gene co-expression network analysis (WGCNA) was performed (706 patients with complete clinical information was available were selected) (Fig. S3a). After adjusting WGCNA parameters, DEGs was divided into 10 modules through average linkage hierarchy clustering (Fig. S3b-e). The blue module containing 190 genes exhibited the highest correlation with NCAPH expression (Pearson’s correlation coefficient = 0.92, *p* < 0.001) (Fig. S3f, g).

### Analysis of potential mechanisms of NCAPH

**Fig. 2:**
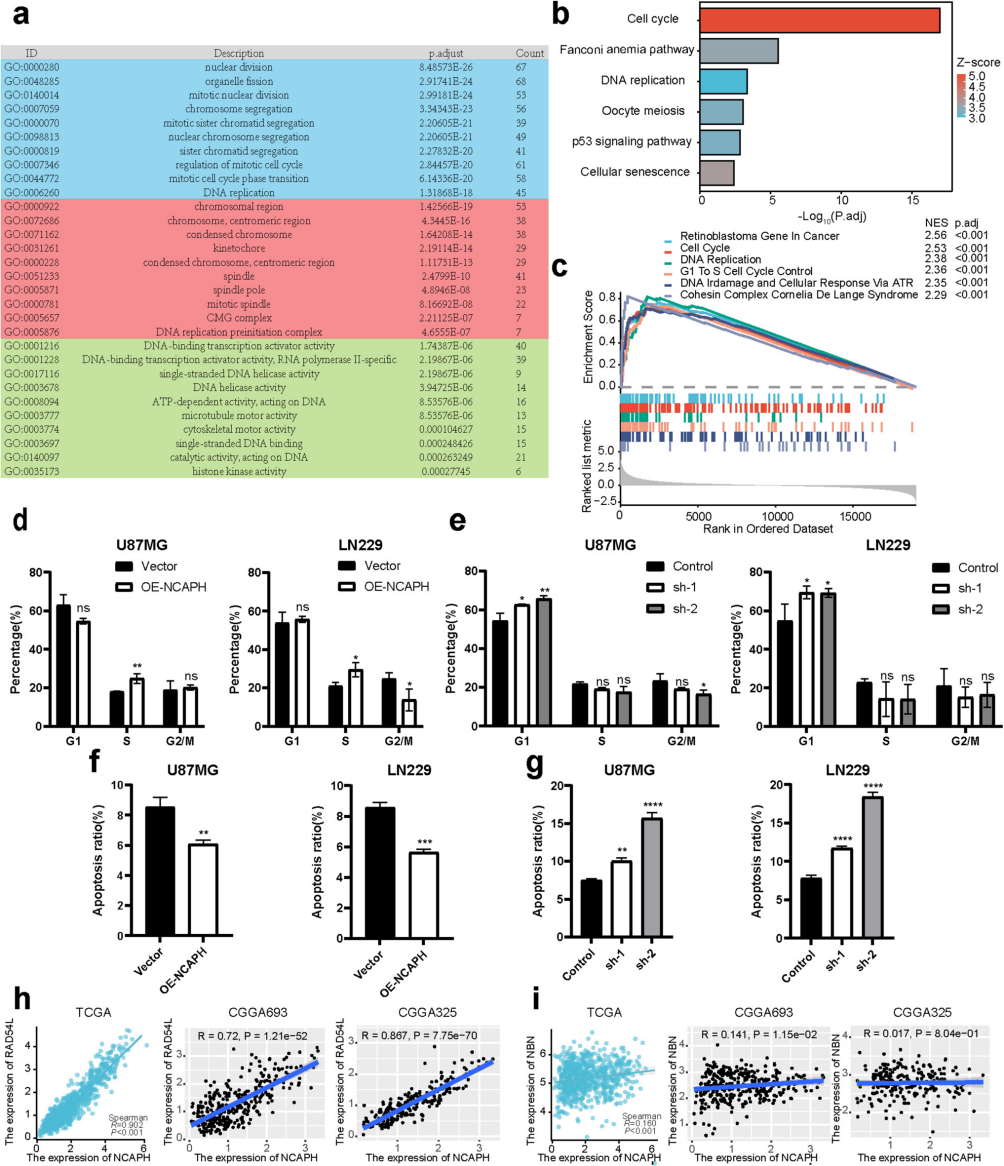
Potential mechanism exploration of NCAPH. **a** GO analysis with blue, red, and green areas representing cell components, biological processes, and molecular functions, respectively. **b** KEGG analysis **c** GSEA of upregulated NCAPH group. **d** Cell cycle in over-expressed NCAPH group. **e** Cell cycle in knockdown NCAPH group. **f** Apoptosis in over-expressed NCAPH group.** g** Apoptosis in knockdown NCAPH group. **h** NCAPH was positively correlated with RAD54L in TCGA database, CGGA693 dataset, and CGGA325 dataset. **i** NCAPH was positively correlated with NBN in TCGA database, CGGA693 dataset, and CGGA325 dataset

Followed by functional enrichment analysis of 190 genes from the blue module. "Nuclear division," "chromosome region," and "DNA-binding transcriptional activator activity" represented the most frequently noted Gene Ontology (GO) terms for the cellular components, biological processes, and the molecular functions, respectively (Fig. 2a). The "cell cycle" was analyzed as the most significant pathway in the Kyoto Encyclopedia of Genes and Genomes (KEGG) analysis (Fig. 2b). Apart from it, results of gene set enrichment analysis (GSEA) of H-NCAPH group consisted of the following aspects: retinoblastoma gene in cancer, cell cycle, DNA replication, G1 to S cell cycle control, DNA damage, and cellular response via ataxia-telangiectasia-mutated-and-Rad3-related Kinase (ATR), Cohesin Complex Cornelia De Lange Syndrome (Fig. 2c). Collectively, NCAPH may regulate tumorigenesis, cell cycle, and DNA replication in glioma.

### Correlation between NCAPH and immune infiltration

As for the role of NCAPH in immunity, results of ESTIMATE analysis indicated that immune and stromal scores were significantly higher in H-NCAPH group, while the tumor purity score of L-NCAPH group was relatively higher (Fig. S5a). It suggested upregulated NCAPH induced immune infiltration in glioma. Subsequently, the analysis of NCAPH and 24 kinds of immune cell infiltration acquired based on ssGSEA algorithm manifested that NCAPH displayed no statistical significance with B cells. And NCAPH negatively correlated with CD8 + T cells, dendritic cells, whereas positively associated with macrophages, neutrophils, and Th2 cells, especially Th2 cells with *R* = 0.898 (*p* < 0.001) (Fig. S5b, c). Based on above findings, NCAPH may play a role in the immune microenvironment of glioma by regulating Th2 cells.

### Overexpression of NCAPH promotes malignant hallmarks

**Fig. 3:**
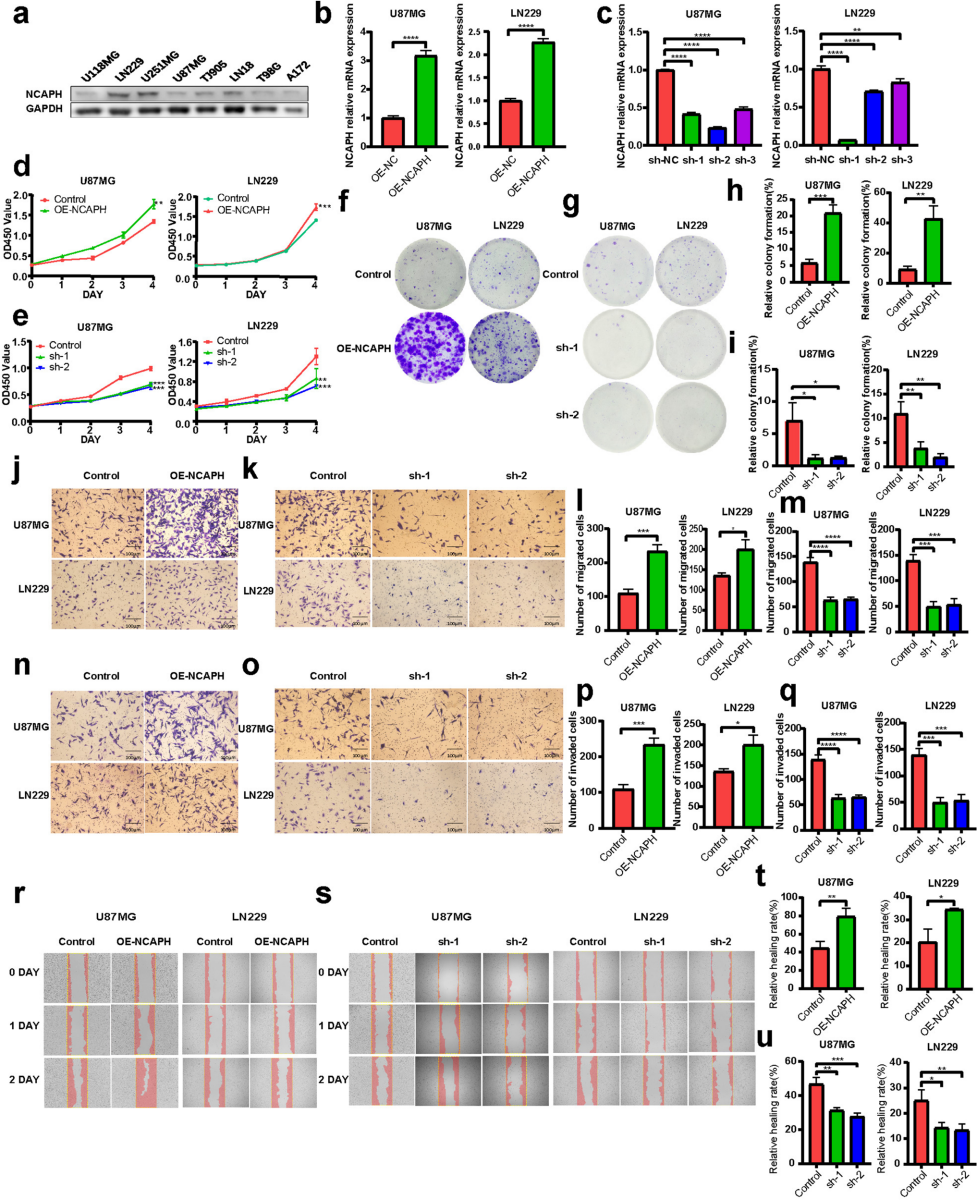
Overexpression of NCAPH promotes proliferation, migration, and invasion of glioma. **a** WB revealed the expression of NCAPH in U118MG, LN229, U251MG, U87MG, TJ905, LN18, T98G, A172 cell lines. **b**,** c** RT-PCR results manifested the transfection efficiency of lentivirus overexpression (OE-NCAPH) and knockdown (shRNA-NCAPH-1, sh-1; shRNA-NCAPH-2, sh-2; shRNA-NCAPH-3, sh-3). **d**,** e** CCK-8 assay indicated that up-regulation and down-regulation of NCAPH could correspondingly promote and reduce the proliferation of glioma cells. **f**,** h** Colony-formation assay revealed upregulated NCAPH improved the colony formation efficiency of glioma cells. **g**,** i** Colony-formation assay results demonstrated the colony formation efficiency of glioma cells was inhibited after NCAPH expression was silenced. **j**–**m** Transwell migration assay showed U87MG and LN229 glioma cells upregulated and down-regulated NCAPH expression in response to migration ability (**j** and **l** were overexpression group, **k** and **m** were knockdown group). The scale bar was 100 μm. **n**–**q** In Transwell invasion assay, the invasion ability of U87MG and LN229 glioma cells after up-regulation and down-regulation of NCAPH was determined (**n** and **p** were overexpression group, **o** and **q** were knockdown group). The scale bar was 100 μm. **r**–**u** Cell wound healing assay manifested the scratch healing ability of cells increased after overexpression of NCAPH (**r**,** t**) while after the expression of NCAPH decreased, the healing ability of the cell scratch decreased (**s**, **u**)

**Fig. 4:**
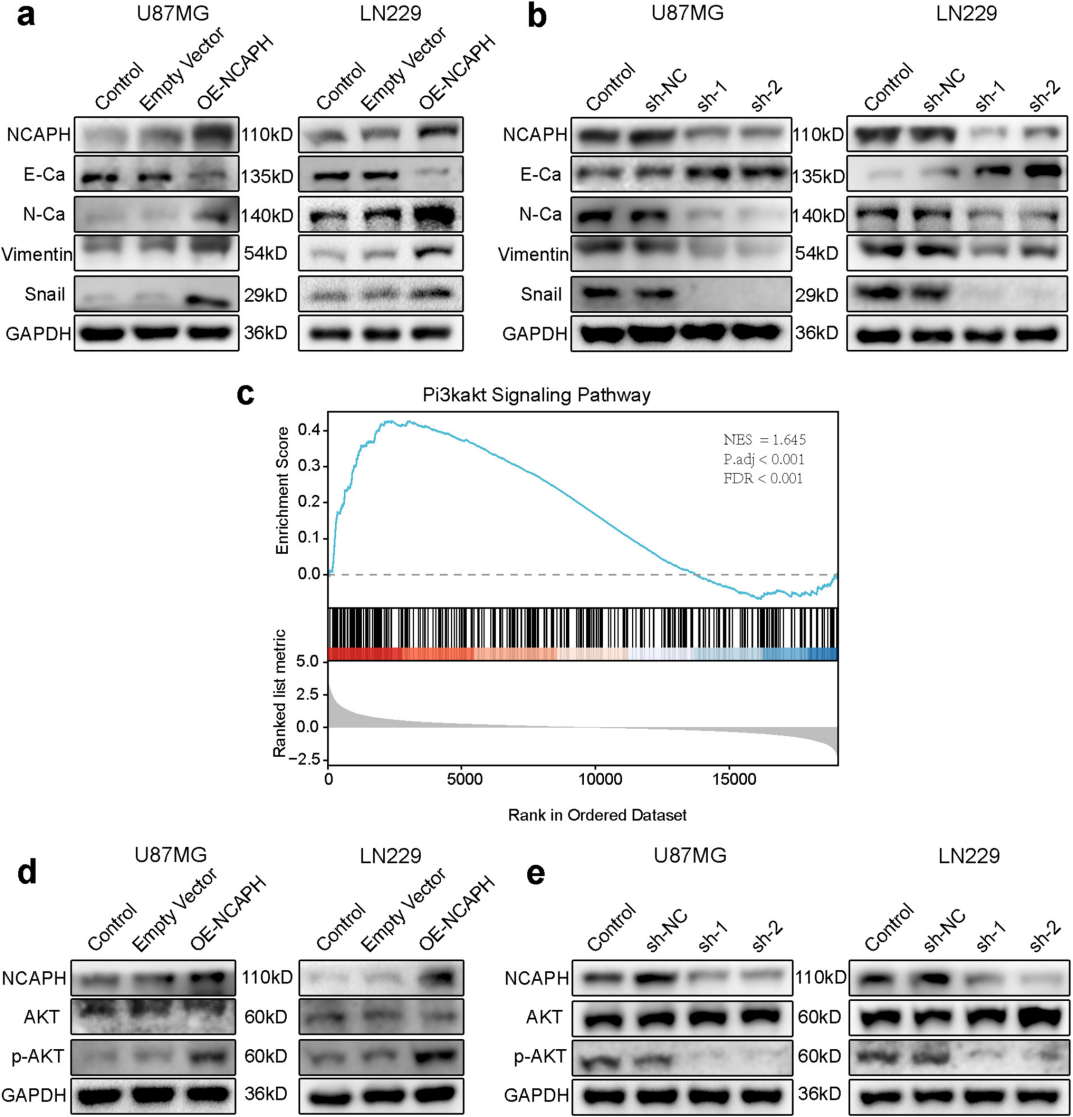
Increased expression of NCAPH promotes the epithelial–mesenchymal transition (EMT) process and produces biological effects through the PI3K/AKT signaling pathway. **a**,** b** WB showed that the expression of NCAPH and EMT markers (E-Ca, N-Ca, vimentin, and Snail) in U87MG and LN229 glioma cells changed after NCAPH overexpression and knockdown. **c** NCAPH-related genes enriched in the PI3K/AKT signaling pathway by GSEA. **d**,** e** The protein levels of NCAPH, AKT, and p-AKT in U87MG and LN229 glioma cells after overexpression and knockdown of NCAPH were detected by WB

Firstly, expression of NCAPH in eight glioma cell lines, including U118MG, U87MG, U251MG, LN229, LN18, T98G, A172, and TJ905, was evaluated by WB. The results identified that the expression of NCAPH was higher in LN229 cell line and lower in U87MG cell line (Fig. 3a) which was the same as results obtained from Human Protein Atlas Database and CCLE database (Fig. S6a, b). According to enrichment analysis results that NCAPH might regulate cell cycle, DNA replication, and tumorigenesis in glioma, we take further experiments to confirm the function of NCAPH. U87MG and LN229 cell lines were taken to establish cell lines with both gain-of-function and loss-of-function of NCAPH by using overexpression virus and three shRNA lentiviruses, respectively. Based on the transfection efficiency confirmed by PCR and WB (Figs. 3b, c and  4a, b), we decided to use shRNA-NCAPH-1 (sh-1) and shRNA-NCAPH-2 (sh-2). Subsequently, the results of flow cytometry revealed upregulated NCAPH increased the proportion of cells in S phase, while reduced the proportion of apoptosis. On the contrary, knockout of NCAPH induced G1 phase cell cycle arrest and promoted cell apoptosis (Fig. 2d, e, f, g) (Fig. S7). In addition, the results of correlation analysis indicated that NCAPH was positively associated with DNA damage repair genes, especially RAD54L (Fig. 2h, i).

Based on the results of flow cytometry, we speculated that NCAPH could regulate the proliferation of glioma cells and CCK-8 assay and Colony-formation assay were performed. Consequently, we observed that in comparison to the control group, the proliferation of transfected OE-NCAPH virus cells was significantly enhanced (*p* < 0.01) (Fig. 3d, f, h) while reduced in shRNA-NCAPH group (*p* < 0.01) (Fig. 3e, g, i).

Subsequently, Transwell assay and Cell wound healing assay were performed to evaluate migration and invasion of glioma cells. The results demonstrated migration ability of glioma cells was enhanced when NCAPH expression was upregulated (Fig. 3j, l), while the mobility reduced in NCAPH knockdown glioma cells (Fig. 3k, m). Then, we attached a Matrigel to the chamber bottom in order to measure Transwell invasion. The findings revealed that overexpression of NCAPH effectively increased the invasive of glioma cells in comparison to the control group (Fig. 3n, p). Conversely, the invasion decreased in down-regulated NCAPH cells (Fig. 3o, q). The results of cell wound healing assay revealed the same results. The healing rate of cell scratch in overexpression group was markedly faster than that in control group (Fig. 3r, t), whereas the healing rate of cell scratch in down-regulated group reduced (Fig. 3s, u).

In conclusion, these results demonstrated that the overexpression of NCAPH promotes malignant hallmarks including DNA damage repair, proliferation, migration, and invasion.

### The upregulated expression of NCAPH promotes EMT process

To elucidate whether NCAPH regulates the migration and invasion of glioma cells via EMT process, we then investigated the expression of EMT-related proteins by WB. The WB results of LN229 and U87MG cells revealed that, in comparison with the control group, the protein expression of different mesenchymal markers (such as N-Ca, Vimentin, and Snail) was increased significantly when NCAPH was upregulated, but the expression of epithelial markers E-Ca was decreased (Fig. 4a). On the contrary, in knockdown of NCAPH group, the expression of epithelial markers E-Ca was increased but several mesenchymal protein markers (including N-Ca, Vimentin, Snail) were significantly decreased (Fig. 4b). These findings indicated that NCAPH regulates mobility of glioma cells by promoting EMT process.

### NCAPH induces malignant hallmarks of glioma cells through modulating activation of PI3K/AKT signaling pathway

In order to elucidate the potential mechanisms of NCAPH regulating glioma cells, 2086 DEGs relevant to NCAPH were obtained for enrichment analysis and enriched in PI3K/AKT signaling pathway (Fig. 4c), which not only involves in the regulation of cell cycle, DNA damage, apoptosis, and cell proliferation but also regulates the invasion and migration of tumor cells [19, 25–28]. Subsequently, WB was performed to test expression of key proteins involved in PI3K/AKT signaling pathway (AKT; Phosphorylation of -AKT (473), p-AKT). The results demonstrated that with the up-regulation of NCAPH expression, the expression of AKT and GAPDH displayed no significant changes, but the p-AKT expression significantly increased (Fig. 4d). Correspondingly, the p-AKT was also decreased after NCAPH was reduced (Fig. 4e). In addition, the results of correlation analysis showed that NCAPH was positively correlated with PDK1 (Fig. S8a-c), the key factor of the PI3K/AKT pathway [34], which regarded as master kinase regulating AGC kinase family members such as AKT, SGK, and S6K [35]. These findings suggested that NCAPH influences malignant hallmarks of glioma cells through activating PI3K /AKT signaling pathway.

**Fig. 5:**
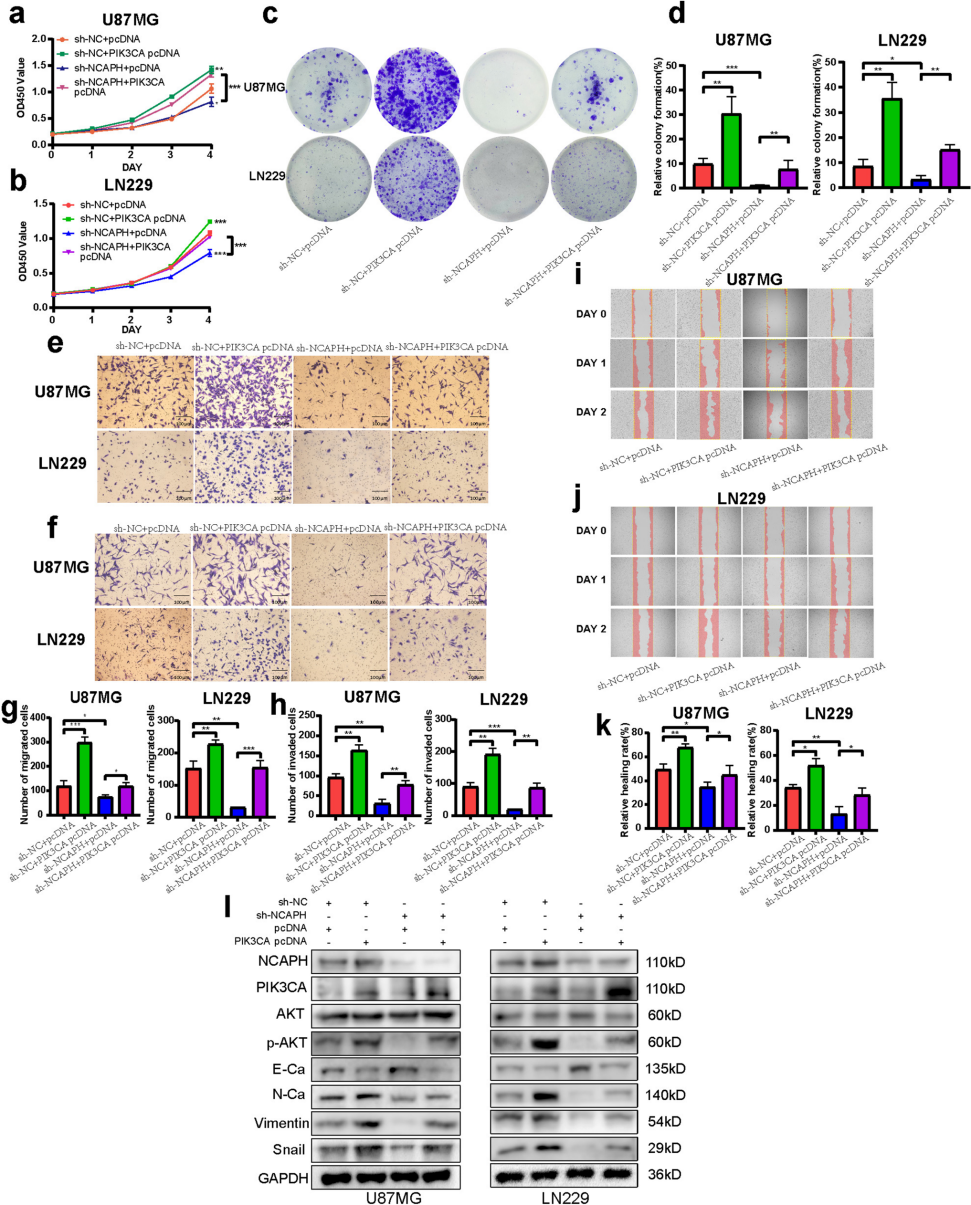
NCAPH promotes proliferation, migration, and invasion of glioma cells by activating PI3K/AKT signaling. **a**,** b** The results of CCK-8 assay reflected the proliferation of sh-NC + pcDNA, sh-NC + PIK3CA pcDNA, sh-NCAPH + pcDNA, and sh-NCAPH + PIK3CA pcDNA in the four groups of glioma cells. **c**,** d** The Colony-formation efficiency of glioma cells was increased after upregulating PIK3CA. **e**–**h** In Transwell assay, PIK3CA overexpression rescued the decline in migration and invasion of glioma cells caused by NCAPH knockdown. **i**–**k** Cell wound healing assay reflected the increased healing rate of cell scratch after up-regulation of PIK3CA. **l** Expression of NCAPH, PIK3CA, AKT, p-AKT, E-Ca, N-Ca, vimentin, and Snail in four groups of glioma cells in WB

To further elucidate the underlying mechanism of the involvement of PI3K/AKT signaling in NCAPH-mediated tumorigenesis of glioma, rescue experiments were used to prove that whether in NCAPH knockdown group, transient overexpression of PIK3CA virus could effectively rescue the proliferation, migration, and invasion ability of glioma cells. The results of CCK-8 assay revealed that although the proliferation ability was significantly reduced after the decrease of NCAPH expression, the proliferation ability of NCAPH knockdown group was improved concomitant with an increase of PIK3CA expression (Fig. 5a, b). This result was also confirmed by Colony-formation assay (Fig. 5c, d). In addition, subsequent Transwell assay also indicated that up-regulation of PIK3CA rescued the reduction in both mobility and invasion in down-regulation NCAPH group (Fig. 5e–h). This observation was also verified by subsequent cell wound healing assay (Fig. 5i–k). Apart from these, WB also identified despite knockdown of NCAPH, overexpression of PIK3CA decreased E-Ca expression along with increase in the Snail, vimentin, and N-Ca expression in glioma cells (Fig. 5l). These findings suggested that NCAPH effectively mediates glioma cell malignant hallmarks and EMT process through promoting the activation of PI3K/AKT signaling pathway.

### Silencing NCAPH inhibits the progression of glioma under in vivo settings

**Fig. 6:**
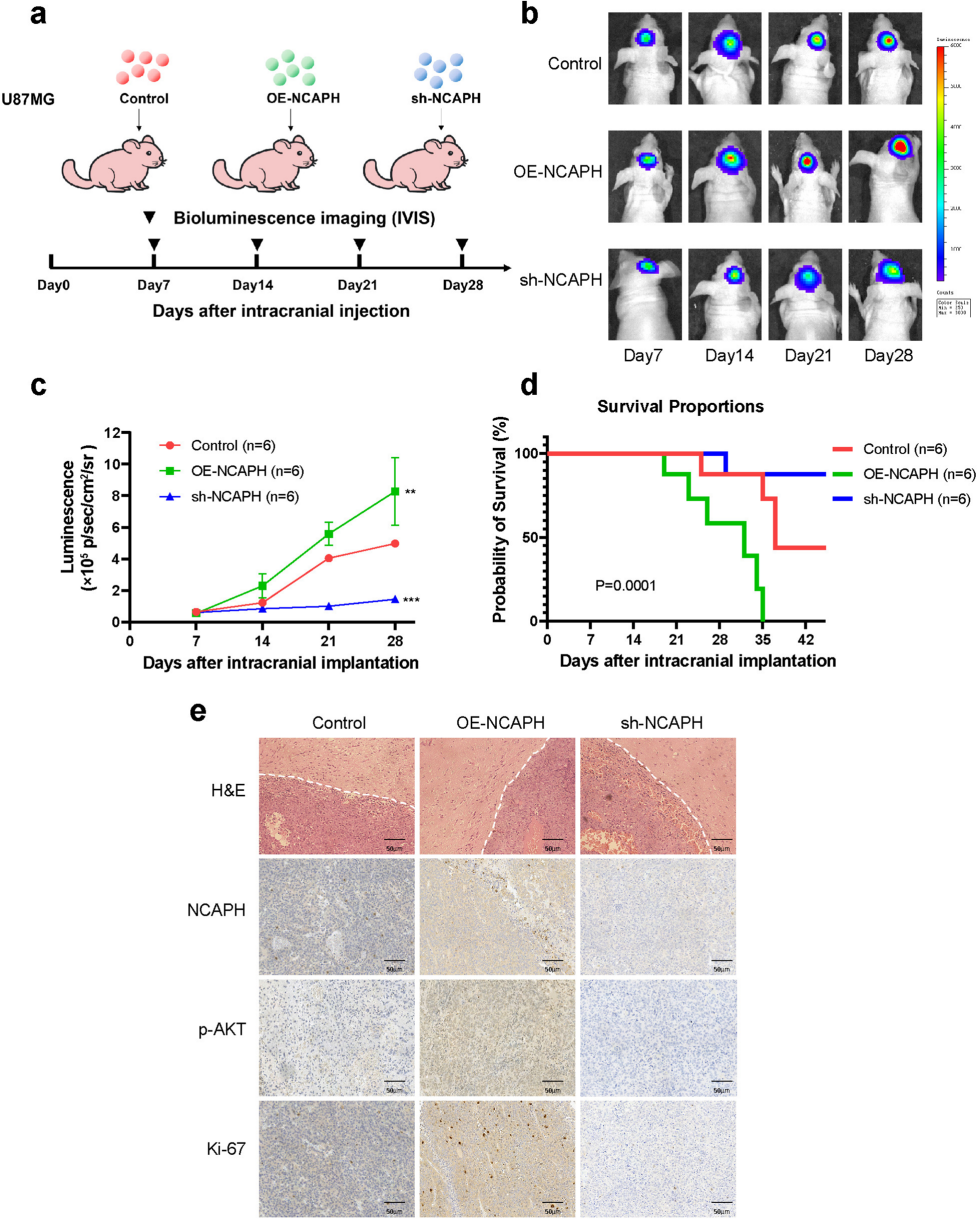
After the silence of NCAPH, the progression of intracranial glioma cells in nude mice was inhibited. **a** Grouping of in vivo experiments, tumor implantation, and bioluminescence imaging. **b** Bioluminescence imaging of intracranial tumor growth. **c** Quantified tumor luminescence signal intensity at week 1, 2, 3, and 4 after implantation. (n = 6 per group). **d** Kaplan–Meier survival curve reflects the survival rate of mice **e** Representative images of H&E staining of the mouse cerebrum showing the tumor border. Immunohistochemical (IHC) staining for NCAPH, p-AKT, and Ki-67 in the samples.

In order to further demonstrate the glioma-promoting effect of NCAPH, intracranial xenotransplantation models were constructed in the nude mice (Fig. 6a). It was observed that in the first week after model construction, intracranial tumors were detected in three groups of nude mouse models. Interestingly, in comparison with the control group, the luminescence of U87MG-OE-NCAPH tumor was found to be significantly higher, and continued to increase in the surrounding monitoring. However, U87MG-shNCAPH tumor grew more slowly over the period of four weeks (Fig. 6b, c). In addition, by analyzing Kaplan–Meier survival curve, we observed that U87MG-shNCAPH tumor bearing mice had a higher survival rate than the control group, but U87MG-OE-NCAPH tumor bearing mice exhibited worse OS (Fig. 6d). H&E staining showed the smaller size tumors in sh-NCAPH group relative to control or OE-NCAPH group. Moreover, we stained the intracranial tumors with NCAPH, p-AKT, and Ki-67 antibodies. The results of IHC analysis revealed decreased NCAPH, p-AKT, and Ki-67 levels in sh-NCAPH group, consistent with the results of the in vitro experiments (Fig. 6e). In vivo experiments further demonstrated NCAPH promotes progression of glioma.

## Discussion

Glioma is the most common primary brain tumor in adults. The main treatment for glioma is surgery, which may be supplemented by radiotherapy and chemotherapy [36, 37]. However, despite these treatments, the prognosis for patients with glioma remains poor. In recent years, new therapies such as CAR-T therapy have emerged but clinical trial results are still unsatisfactory [8–14]. Therefore, we are trying to find a new molecular therapeutic target for glioma treatment.

NCAPH, a subunit of condensin I, plays an important role in the accurate segregation of sister chromatids during the maintenance of mitosis[15–17]. NCAPH has been shown to promote the malignant progression of many cancers, such as breast cancer [38], bladder cancer [24], and colorectal cancer [20]. Our study found that NCAPH is highly expressed in gliomas and associated with poor prognosis.

Molecular diagnosis was added to the diagnosis and treatment of gliomas in 2008 [39]. IDH mutation mainly occur in low-grade gliomas and secondary GBM. Importantly, IDH wild-type is associated with poor clinical outcomes in glioma patients [6]. Our study showed that high expression of NCAPH corresponded to worse pathophysiological features of gliomas, such as higher WHO grade, IDH wild-type, and non-codeletion of 1p/19q. These findings suggest that NCAPH plays an important role in the malignant development of glioma.

NCAPH has a pro-tumor effect. NCAPH can promote the proliferation and mobility of various cancer cells through PI3K/AKT and MEK/ERK signaling pathways [19, 24, 38, 40]. Glioma cells are characterized by less apoptosis and higher proliferation and metastatic capacity[41, 42]. However, the function of NCAPH in glioma remains unclear. Our study is the first to demonstrate NCAPH promotes malignant hallmarks of glioma. The results of CCK-8 experiment and Colony-formation assay revealed that the upregulated NCAPH could promote the proliferation of glioma cells. In addition, Transwell assay and Cell wound healing assay manifested that the overexpression of NCAPH could enhance the migration and invasion ability of glioma cells. Besides, enrichment analysis showed that NCAPH-related genes were associated with cell cycle, DNA replication, and nuclear division, among which NCAPH promoted cell cycle from G1 to S phase. The results of flow cytometry identified that when NCAPH was down-regulated, the apoptosis of glioma cells increased and they were arrested in G1 phase. Results are consistent with conclusions drawn from studies on gastric cancer and breast cancer [21, 22]. In correlation analysis, NCAPH was positively correlated with DNA damage repair genes suggesting that decreased NCAPH may induce DNA damage in glioma cells. It is worth noting that the principle of radiotherapy and chemotherapy drugs after glioma surgery is to disrupt the DNA replication of glioma cells. For example, temozolomide, the most commonly used drug in clinic, achieves tumor killing function by inducing DNA strand breaks and damage in tumor cells [43, 44]. Besides, NCAPH is positively correlated with the level of acquired immune cells (Th2 cells) in lung adenocarcinoma[45]. We also obtained the same conclusion in the immune infiltration analysis of NCAPH and glioma. Inhibition of Th2 cells may help to prevent the spread, recurrence, and metastasis of cancer cells [46]. These results demonstrated that targeting NCAPH may be a potential therapeutic approach to inhibit glioma progression in the future.

The pathway that NCAPH may regulate in glioma remains unknown. Therefore, we further performed GSEA and found that NCAPH-related genes were highly associated with PI3K/AKT signaling pathway. In various cancers, the PI3K/AKT pathway promotes tumor proliferation, migration, invasion, and EMT process [34, 47]. In cervical cancer and breast cancer, NCAPH can induce the tumorigenesis through PI3K/AKT pathway [19, 38]. In addition, in pediatric high-grade gliomas and adult glioblastomas, the use of PI3K inhibitors enhances the sensitivity of DNA damage therapy to tumors [29, 48]. The results of WB showed that p-AKT upregulated after NCAPH increased. Importantly, overexpression of PIK3CA in glioma cells rescued knockdown NCAPH mediated proliferation, migration, invasion, and EMT processes. Moreover, based on the blue module genes screened by WGCNA and existing researches, AURKA, TPX2, CHEK1, and PLK1 were found to be highly related to NCAPH (Fig. S4) and were involved in the regulation of PI3K/AKT pathway in cancer [49–52]. Therefore, we speculated that these four genes may be the downstream targets of NCAPH. These findings indicate that NCAPH promotes malignant progression in glioma by activating PI3K/AKT pathway.

Despite these findings, our exploration of NCAPH has some limitations. Firstly, the downstream targets of NCAPH in glioma have only been briefly analyzed, and the more specific mechanism of NCAPH in regulating PI3K/AKT pathway is still unknown. Second, there is a lack of drugs that target NCAPH. Therefore, more experiments are needed in the future to determine the further mechanism by which NCAPH regulates the PI3K/AKT pathway. Meanwhile, the development of fat-soluble drugs targeting NCAPH may be a promising direction.

In conclusion, our study reveals the correlation between NCAPH and the molecular and clinical characterization in glioma. NCAPH is closely associated with poor prognosis of glioma patients. We firstly identified NCAPH promotes the proliferation, migration, invasion, and EMT process of glioma cells via PI3K/AKT pathway, and NCAPH is positively correlated with DNA damage repair in glioma cells. Thus, NCAPH can serve as a novel diagnostic marker for glioma progression and a potential therapeutic target in the future.

## Supplementary Information

Below is the link to the electronic supplementary material.

Supplementary material 1 (TIF 1104 kb)

Supplementary material 2 (TIF 6544 kb)

Supplementary material 3 (TIF 2741 kb)

Supplementary material 4 (TIF 1500 kb)

Supplementary material 5 (TIF 1116 kb)

Supplementary material 6 (TIF 853 kb)

Supplementary material 7 (TIF 1714 kb)

Supplementary material 8 (TIF 481 kb)

Supplementary material 9 (DOCX 1912 kb)

Supplementary material 10 (DOCX 15 kb)

Supplementary material 11 (DOCX 15 kb)

## Data Availability

Data are available in public repository, including CGGA (http://www.cgga.org.cn), TCGA (https://xenabrowser.net), GTEx (https://xenabrowser.net), and The Human Protein Atlas (https://www.proteinatlas.org).
