# Supplementary figures and images for: NCAPH serves as a prognostic factor and promotes the tumor progression in glioma through PI3K/AKT signaling pathway
Source: Mol Cell Biochem. 2024 Apr 8;480(1):589–605. doi: 10.1007/s11010-024-04976-4 (PMC11695388; doi:10.1007/s11010-024-04976-4)

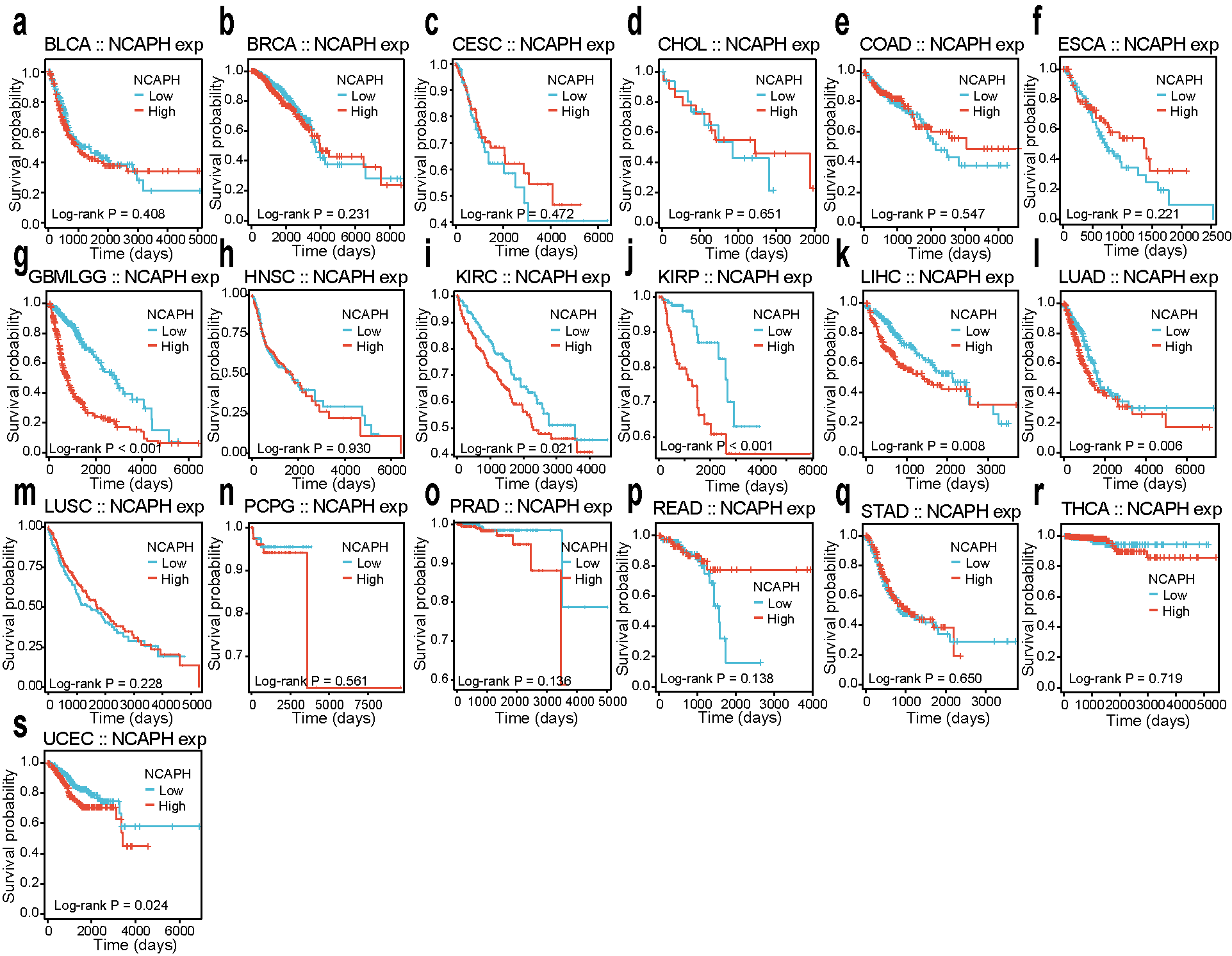

Supplement: Supplementary file 1 — Supplementary material 1 (TIF 1104 kb) [file 11010_2024_4976_MOESM1_ESM.tif]

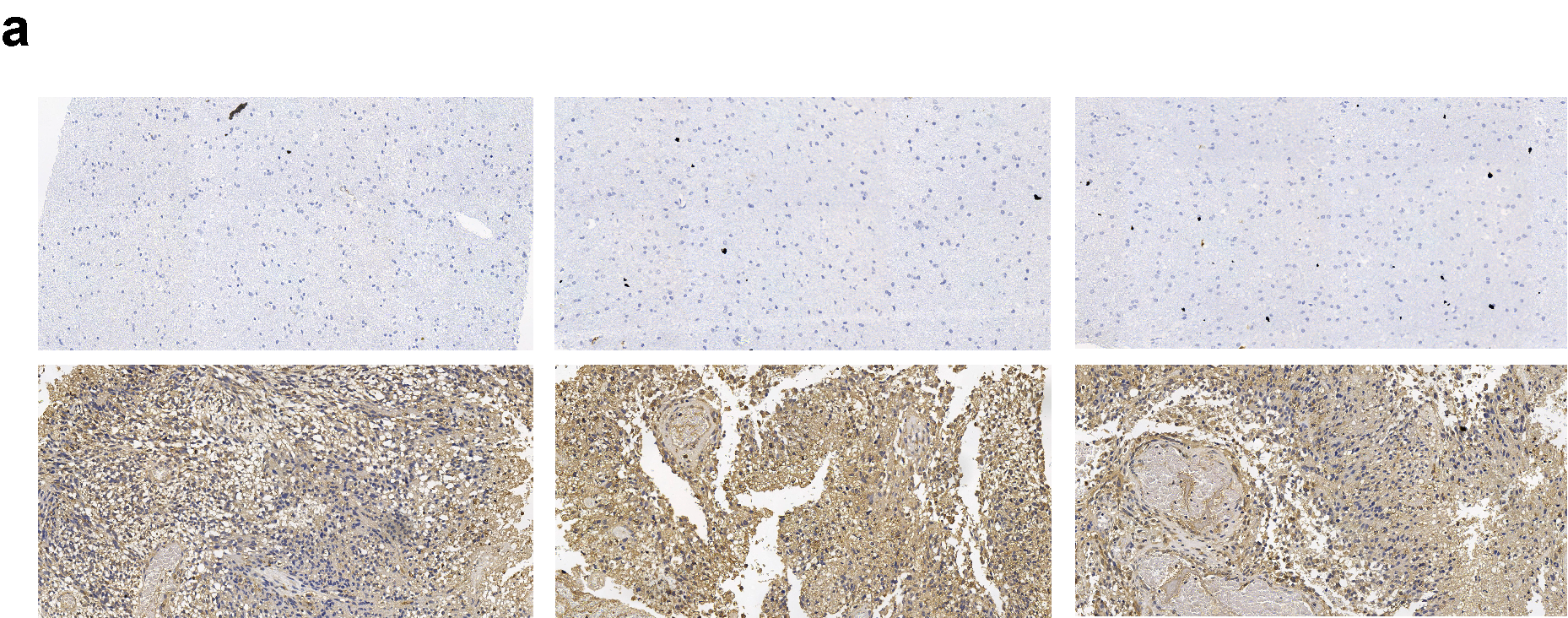

Supplement: Supplementary file 2 — Supplementary material 2 (TIF 6544 kb) [file 11010_2024_4976_MOESM2_ESM.tif]

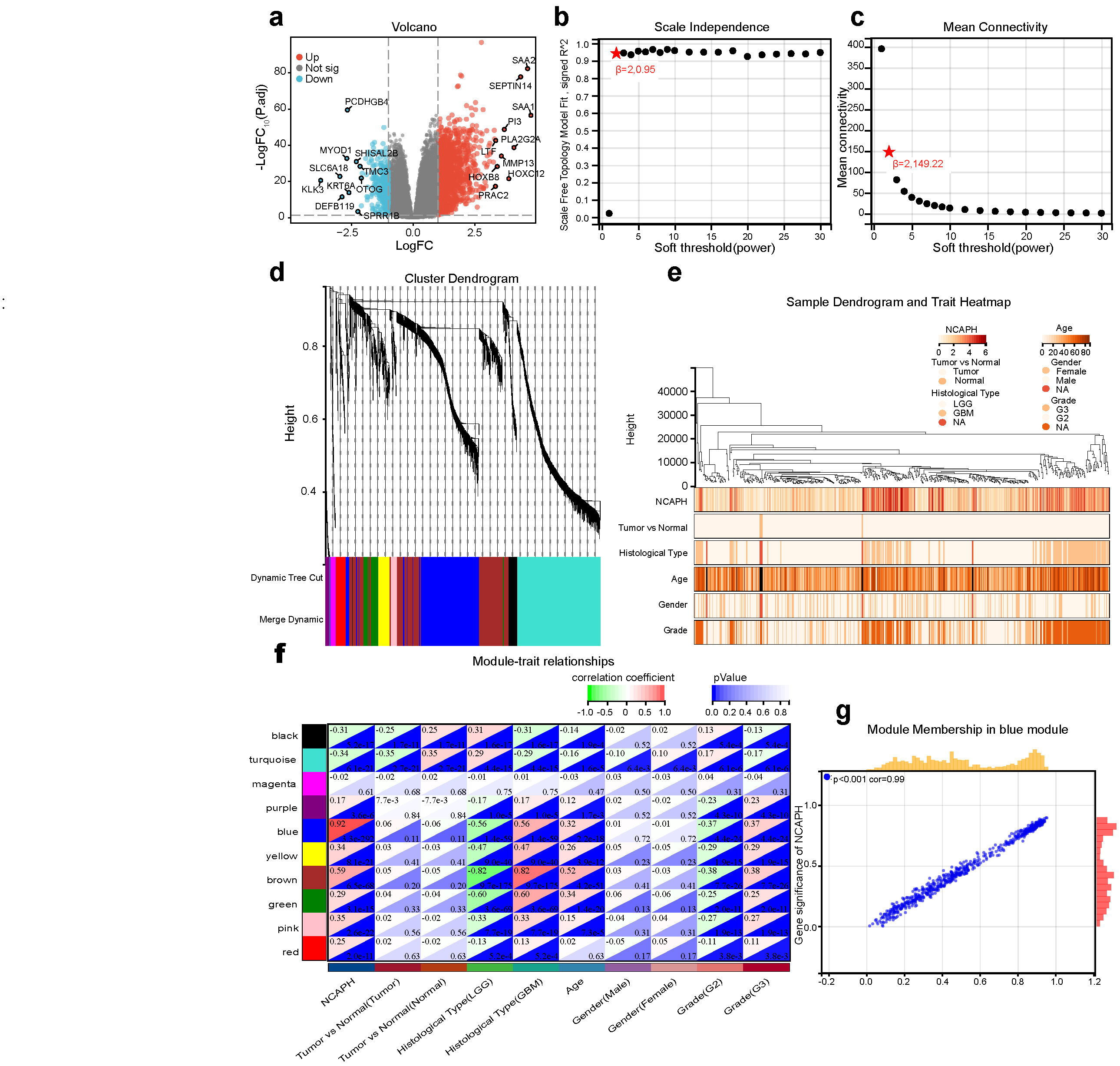

Supplement: Supplementary file 3 — Supplementary material 3 (TIF 2741 kb) [file 11010_2024_4976_MOESM3_ESM.tif]

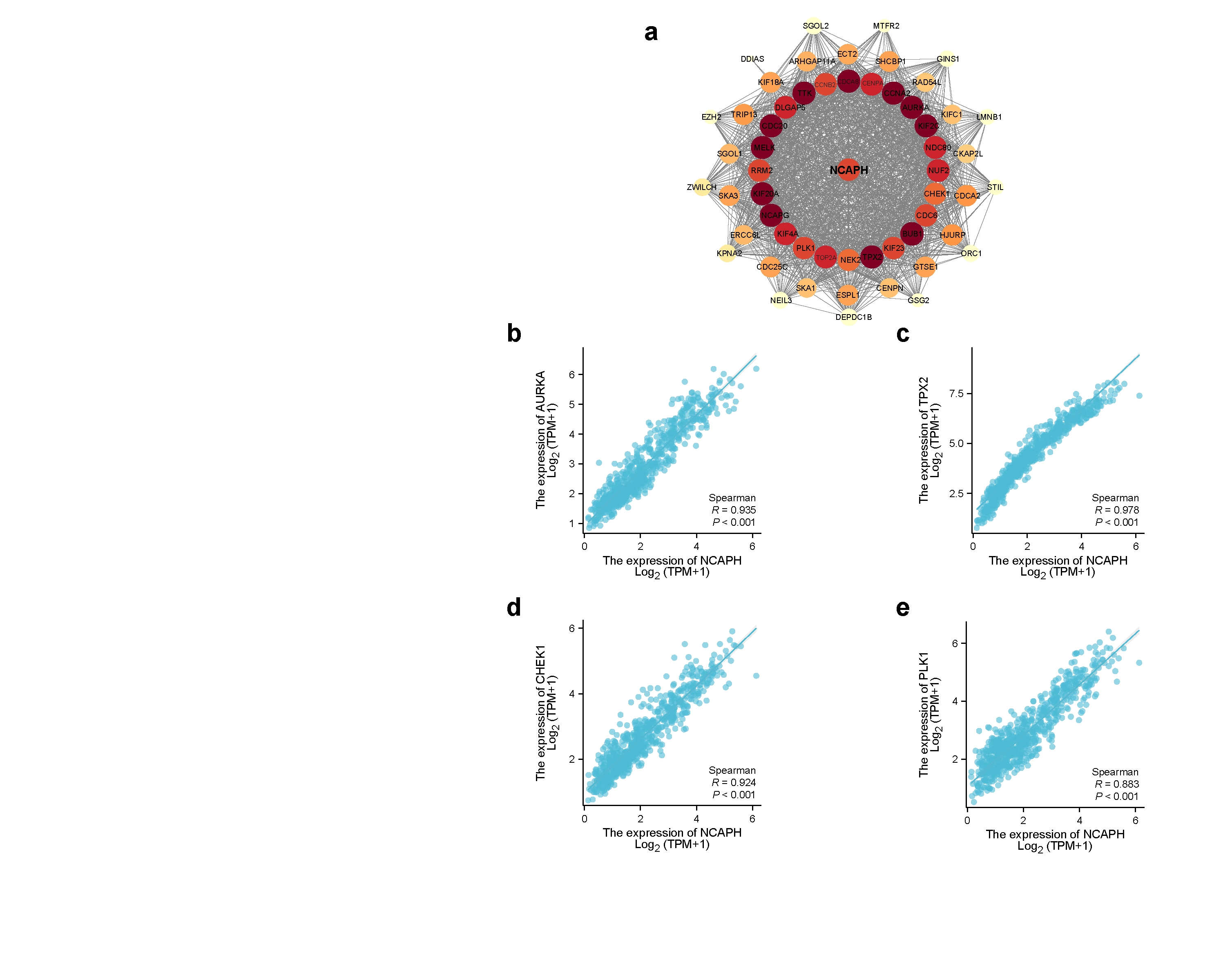

Supplement: Supplementary file 4 — Supplementary material 4 (TIF 1500 kb) [file 11010_2024_4976_MOESM4_ESM.tif]

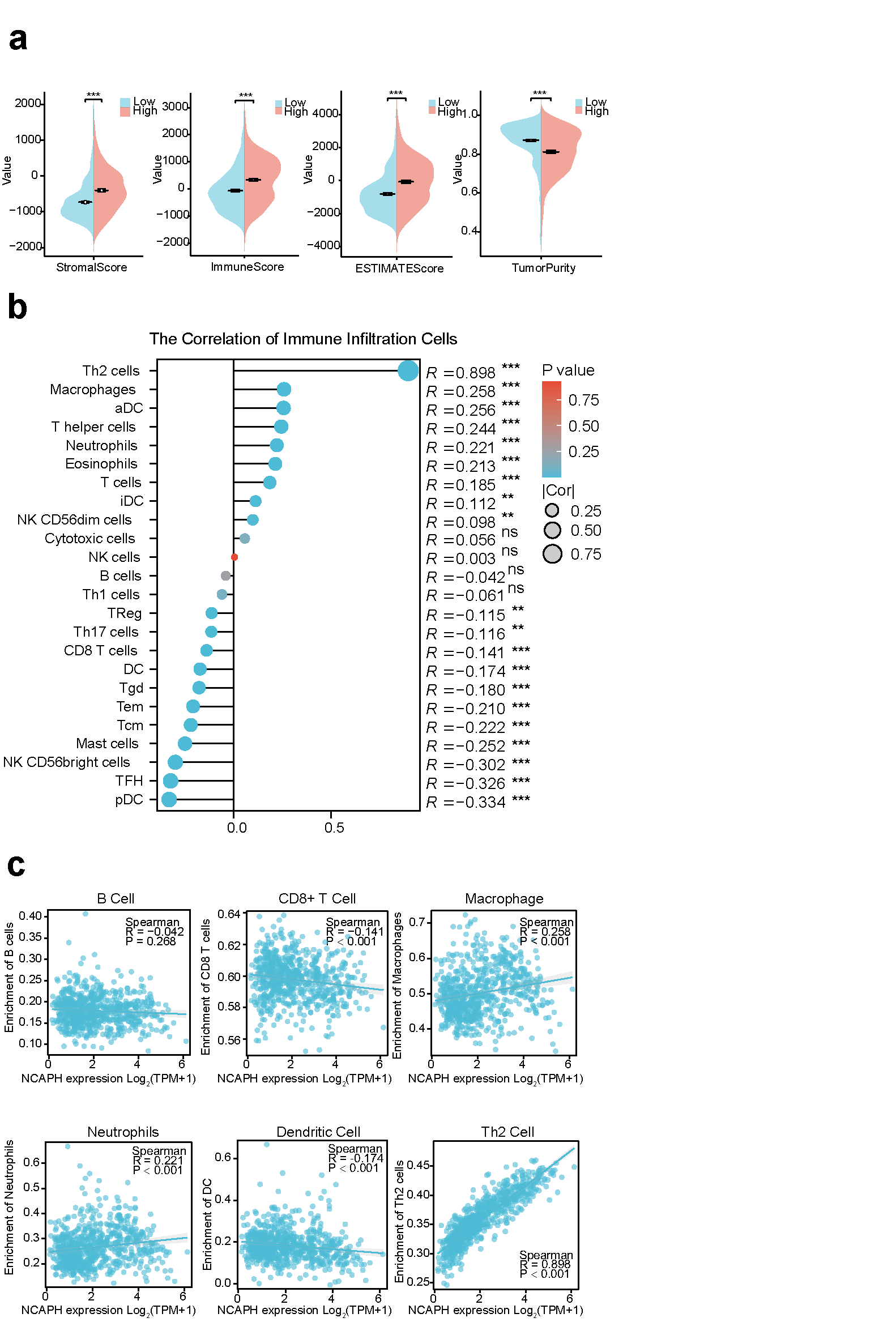

Supplement: Supplementary file 5 — Supplementary material 5 (TIF 1116 kb) [file 11010_2024_4976_MOESM5_ESM.tif]

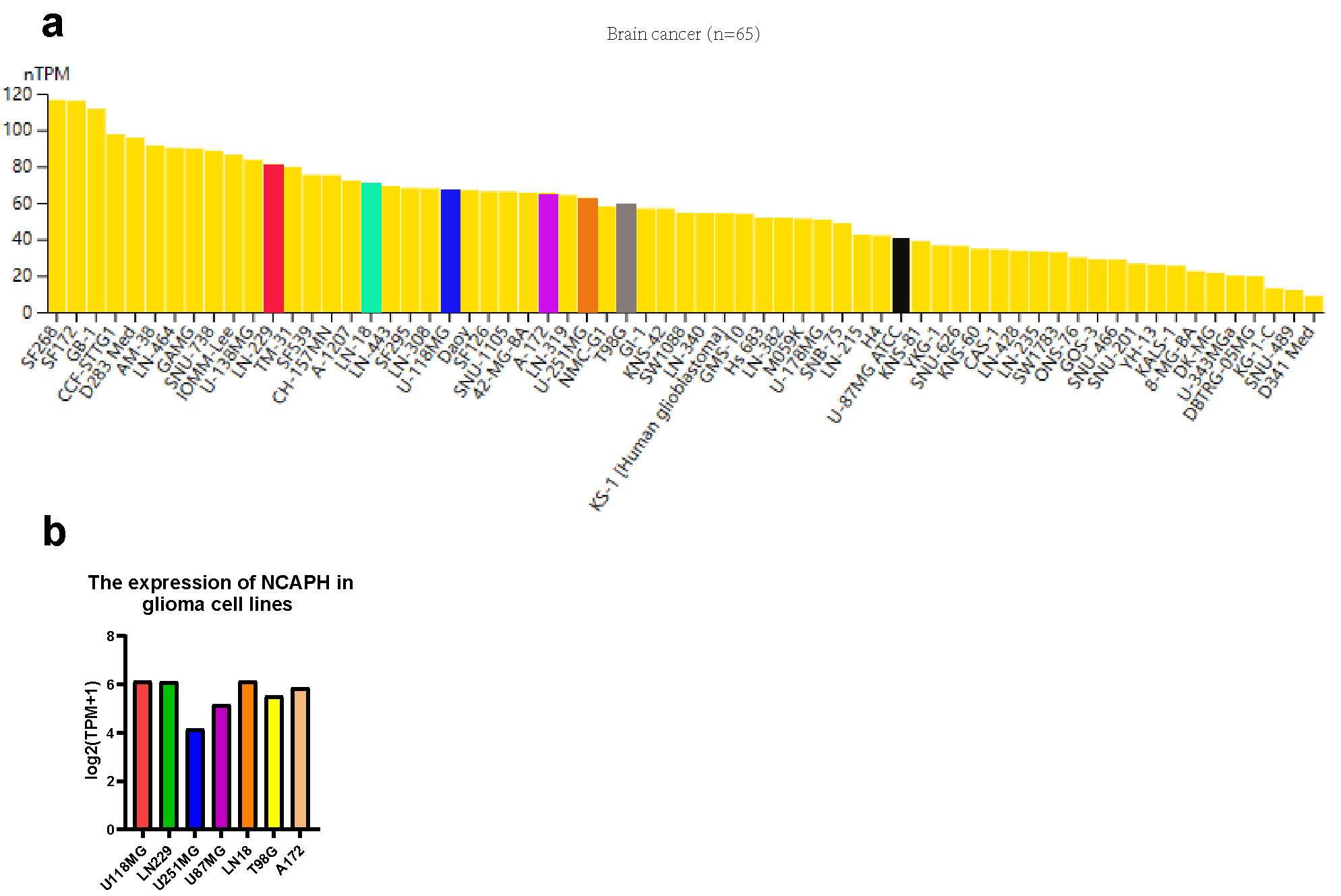

Supplement: Supplementary file 6 — Supplementary material 6 (TIF 853 kb) [file 11010_2024_4976_MOESM6_ESM.tif]

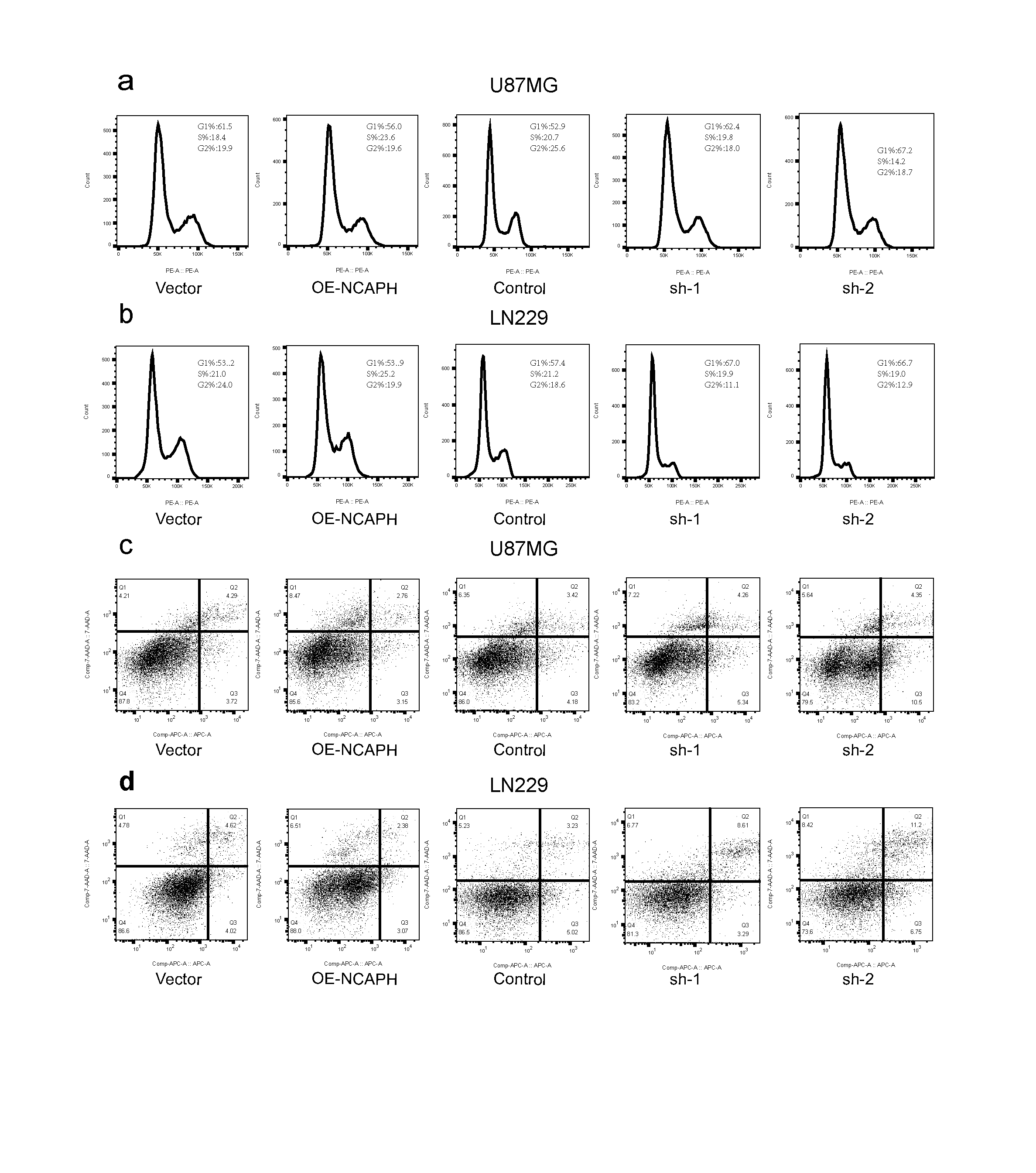

Supplement: Supplementary file 7 — Supplementary material 7 (TIF 1714 kb) [file 11010_2024_4976_MOESM7_ESM.tif]

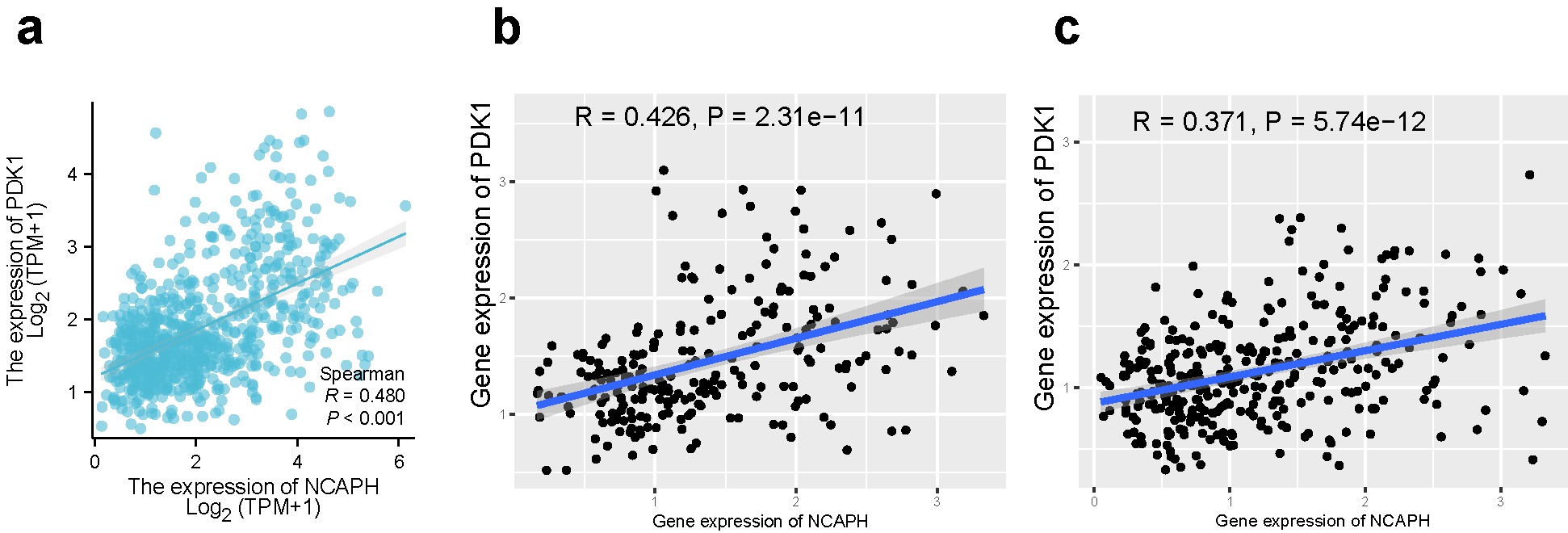

Supplement: Supplementary file 8 — Supplementary material 8 (TIF 481 kb) [file 11010_2024_4976_MOESM8_ESM.tif]
