# Supplementary material for: NCAPH serves as a prognostic factor and promotes the tumor progression in glioma through PI3K/AKT signaling pathway
Source: Mol Cell Biochem. 2024 Apr 8;480(1):589–605. doi: 10.1007/s11010-024-04976-4 (PMC11695388; doi:10.1007/s11010-024-04976-4)
Supplement: Supplementary file 9 — Supplementary material 9 (DOCX 1912 kb) [file 11010_2024_4976_MOESM9_ESM.docx]

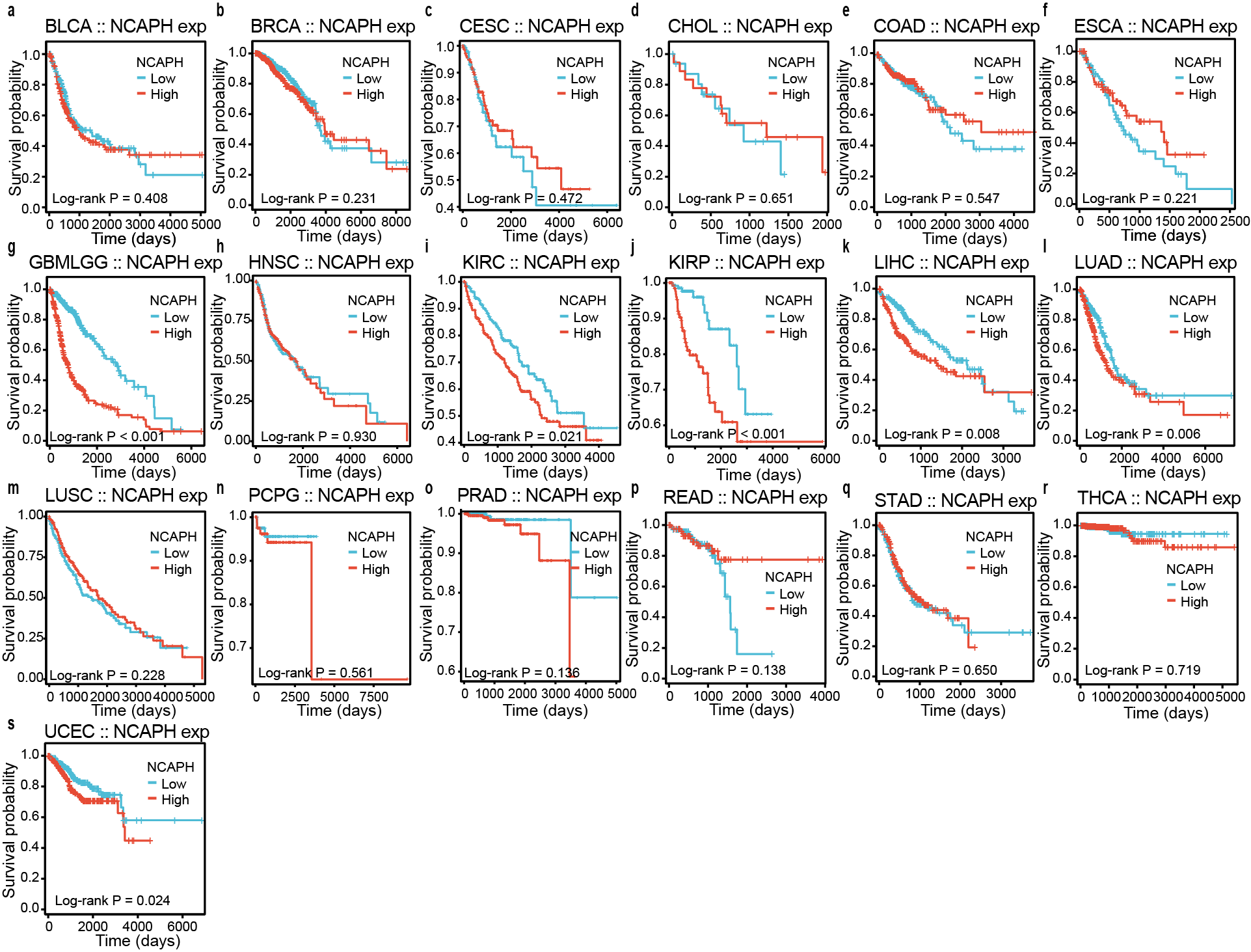


**Fig.S1 Clinical value of NCAPH mRNA in pan-cancer.** **a-s** K-M survival analysis in 19 cancer types was utilized to identified respectively differences in overall survival (OS) between the H-NCAPH and L-NCAPH groups. (**a**, BLCA; **b**, BRCA; **c**, CESC; **d**, CHOL; **e**, COAD; **f**, ESCA; **g**, GAMLGG; **h**, HNSC; **i**, KIRC; **j**, KIRP; **k**, LIHC; **l**, LUAD; **m**, LUSC; **n**, PCPG; **o**, PRAD; **p**, READ; **q**, STAD; **r**, THCA; **s**, UCEC) (^ns^p>0.05, *p<0.05, **p<0.01, ***p<0.001, **** P<0.0001 here and in the following figures).


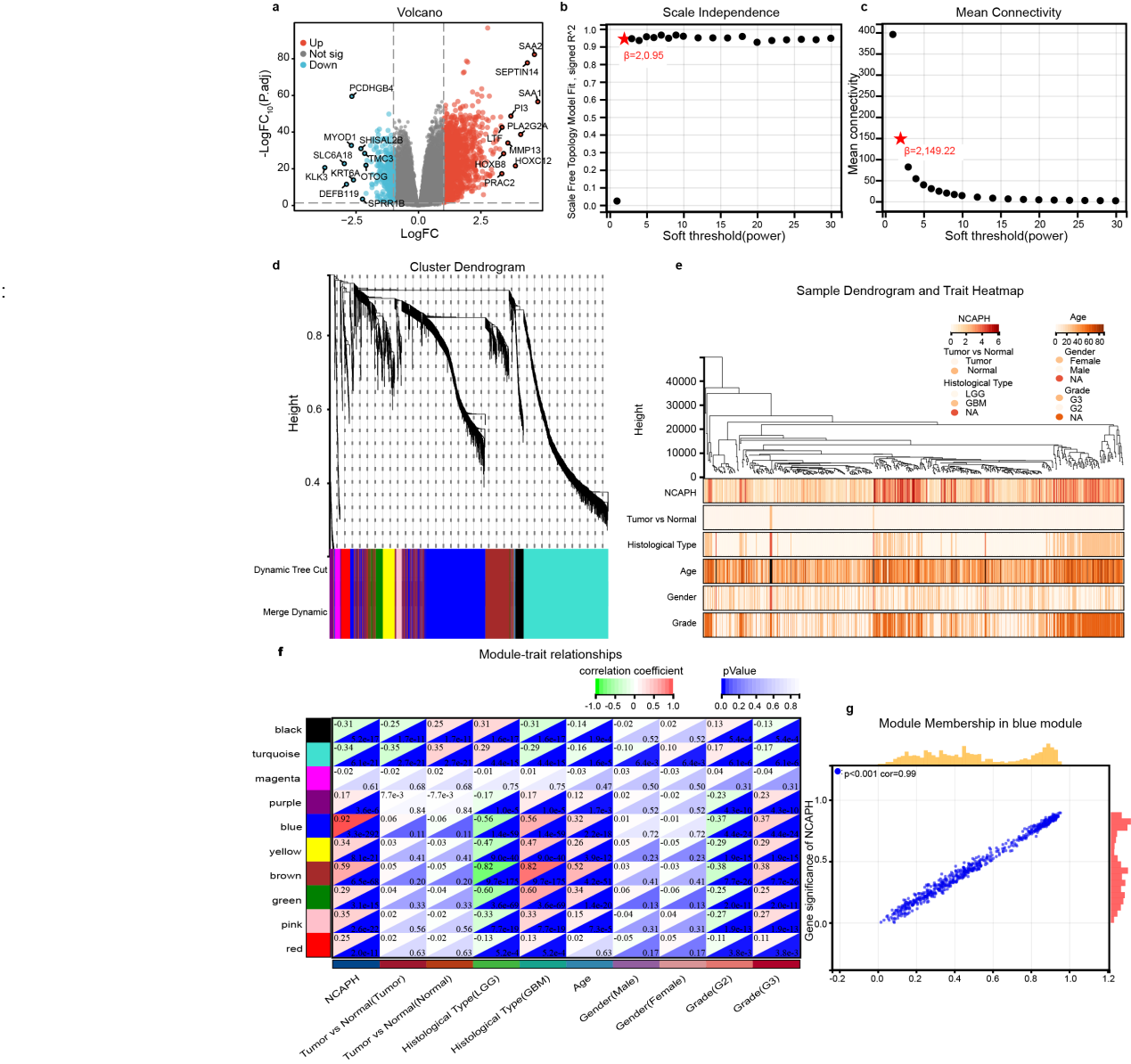


**Fig.S2 Screening of NCAPH-related modules and genes. a** Volcano map between glioma and adjacent tissues based on DEG in TCGA database. **b** Calculation of scale-free fitting index of various soft threshold powers. **c** Analysis of the average connectivity of various soft-thresholding powers. **d** Cluster dendrogram of 706 glioma patients. **e** Cluster 2086 DEGs based on the difference measure (1-TOM) and divide them into 10 modules. **f** The correlation heatmap between module characteristic genes and glioma clinical parameters (NCAPH played as the main study object). **g** The scatter map of blue module characteristic genes.


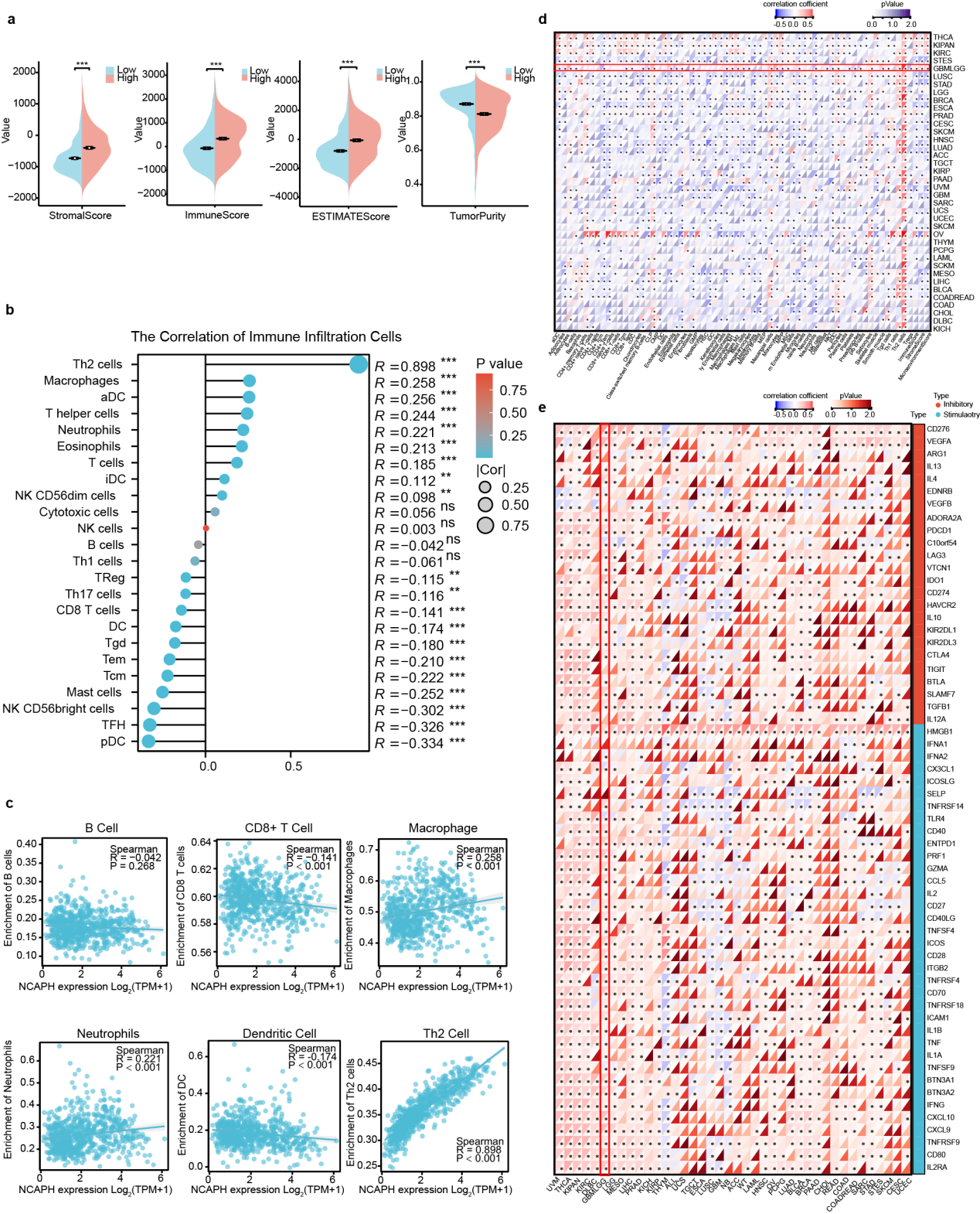


**Fig.S3 The glioma cells analysis of NCAPH and immune infiltration. a** Measures of stromal scores, immune scores, ESTIMATE scores, and tumor purity between the Estimation-based L-NCAPH and H-NCAPH groups. **b** The bubble gum plot revealed the correlation between NCAPH and immune cell infiltration in glioma. **c** The correlated analysis between NCAPH and B cells, CD8+T cells, macrophages, neutrophils, dendritic cells and Th2 cells. **d** Heat map of correlation between NCAPH and immune cell infiltration in pan-cancer. **e** Heat map of correlation between NCAPH and immune checkpoint in pan-cancer.


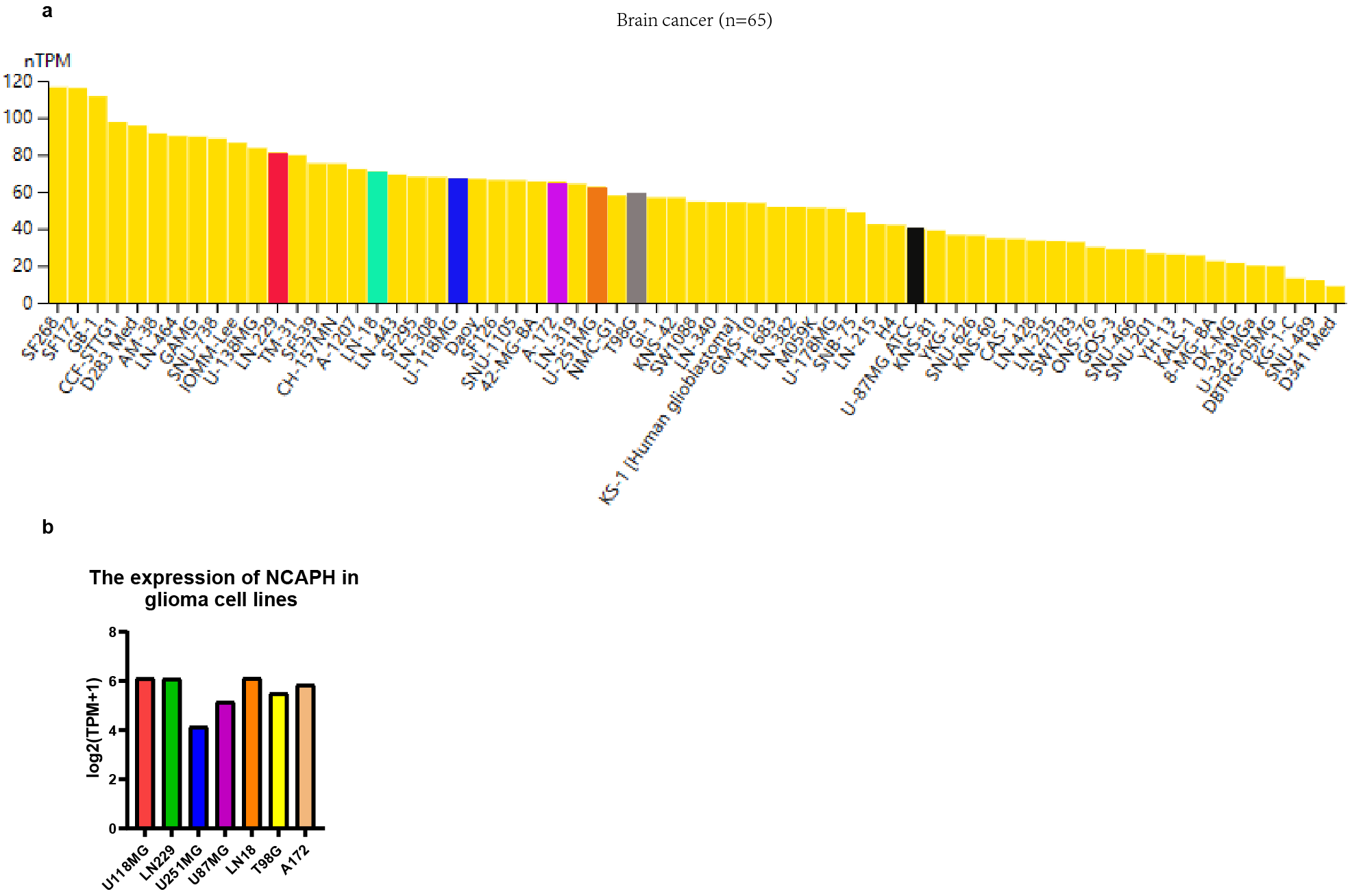


**Fig.S4 NCAPH expression in different cell lines of glioma from diverse databases**

**a** NCAPH expression of glioma cell lines in the Human Protein Atlas database.

**b** NCAPH expression of glioma cell lines in CCLE database.
