# Supplementary material for: NCAPH serves as a prognostic factor and promotes the tumor progression in glioma through PI3K/AKT signaling pathway
Source: Mol Cell Biochem. 2024 Apr 8;480(1):589–605. doi: 10.1007/s11010-024-04976-4 (PMC11695388; doi:10.1007/s11010-024-04976-4)
Supplement: Supplementary file 10 — Supplementary material 10 (DOCX 15 kb) [file 11010_2024_4976_MOESM10_ESM.docx]

**Table S1 The clinical relevance analysis of NCAPH expression in patients with gliomas**

| Characteristics | Low expression of NCAPH | High expression of NCAPH | P value |
| --- | --- | --- | --- |
| n | 349 | 350 |  |
| Age, n (%) |  |  | < 0.001 |
| <= 60 | 310 (44.3%) | 246 (35.2%) |  |
| > 60 | 39 (5.6%) | 104 (14.9%) |  |
| Gender, n (%) |  |  | 0.9740 |
| Male | 200 (28.6%) | 201 (28.8%) |  |
| Female | 149 (21.3%) | 149 (21.3%) |  |
| WHO grade, n (%) |  |  | < 0.001 |
| G2 | 182 (28.6%) | 42 (6.6%) |  |
| G3 | 111 (17.4%) | 134 (21%) |  |
| G4 | 15 (2.4%) | 153 (24%) |  |
| Histological type, n (%) |  |  | < 0.001 |
| Astrocytoma | 113 (16.2%) | 83 (11.9%) |  |
| Oligoastrocytoma | 91 (13%) | 44 (6.3%) |  |
| Oligodendroglioma | 130 (18.6%) | 70 (10%) |  |
| Glioblastoma | 15 (2.1%) | 153 (21.9%) |  |
| IDH status, n (%) |  |  | < 0.001 |
| WT | 52 (7.5%) | 194 (28.2%) |  |
| Mut | 293 (42.5%) | 150 (21.8%) |  |
| 1p/19q codeletion, n (%) |  |  | < 0.001 |
| Non-codel | 228 (32.9%) | 292 (42.2%) |  |
| Codel | 120 (17.3%) | 52 (7.5%) |  |
