# Supplementary material for: NCAPH serves as a prognostic factor and promotes the tumor progression in glioma through PI3K/AKT signaling pathway
Source: Mol Cell Biochem. 2024 Apr 8;480(1):589–605. doi: 10.1007/s11010-024-04976-4 (PMC11695388; doi:10.1007/s11010-024-04976-4)
Supplement: Supplementary file 11 — Supplementary material 11 (DOCX 15 kb) [file 11010_2024_4976_MOESM11_ESM.docx]

**Table S2** **Univariate and multivariate analysis of NCAPH**

| Characteristics | Total(N) | Univariate analysis | |  | Multivariate analysis | |
| --- | --- | --- | --- | --- | --- | --- |
|  |  | Hazard ratio (95% CI) | P value |  | Hazard ratio (95% CI) | P value |
| Age | 604 | 1.075 (1.063 - 1.088) | **< 0.001** |  | 1.052 (1.038 - 1.066) | **< 0.001** |
| Gender | 604 |  | 0.956 |  |  |  |
| female | 252 | Reference |  |  |  |  |
| male | 352 | 1.008 (0.750 - 1.357) | 0.956 |  |  |  |
| Grade | 604 |  | **< 0.001** |  |  |  |
| ≤G2 | 214 | Reference |  |  | Reference |  |
| >G2 | 390 | 6.132 (3.909 - 9.617) | **< 0.001** |  | 2.409 (1.458 - 3.979) | **< 0.001** |
| IDH status | 598 |  | **< 0.001** |  |  |  |
| Mutant | 375 | Reference |  |  | Reference |  |
| WT | 223 | 10.774 (7.582 - 15.310) | **< 0.001** |  | 3.760 (2.340 - 6.041) | **< 0.001** |
| 1p/19q codeletion | 598 |  | **< 0.001** |  |  |  |
| non-codel | 448 | Reference |  |  | Reference |  |
| codel | 150 | 0.220 (0.129 - 0.374) | **< 0.001** |  | 0.479 (0.259 - 0.884) | **0.019** |
